# Synthesis, Characterization, and Biological Evaluation of Tetrahydropyrimidines: Dual-Activity and Mechanism of Action

**DOI:** 10.3390/pharmaceutics14102254

**Published:** 2022-10-21

**Authors:** Emilija Milović, Nenad Janković, Jelena Petronijević, Nenad Joksimović, Marijana Kosanić, Tatjana Stanojković, Ivana Matić, Nađa Grozdanić, Olivera Klisurić, Srđan Stefanović

**Affiliations:** 1 University of Kragujevac, Institute for Information Technologies Kragujevac, Department of Sciences, Jovana Cvijića bb, 34000 Kragujevac, Serbia; 2 University of Kragujevac, Faculty of Science, Department of Chemistry, Radoja Domanovića 12, 34000 Kragujevac, Serbia; 3 University of Kragujevac, Faculty of Science, Department of Biology and Ecology, Radoja Domanovića 12, 34000 Kragujevac, Serbia; 4 Institute of Oncology and Radiology of Serbia, Pasterova 14, 11000 Belgrade, Serbia; 5 University of Novi Sad, Faculty of Science, Department of Physics, Trg Dositeja Obradovića 3, 21000 Novi Sad, Serbia; 6 Institute of Meat Hygiene and Technology, Kaćanskog 13, 11000 Belgrade, Serbia

**Keywords:** Biginelli reaction, tetrahydropyrimidine, antimicrobial, anticancer, apoptosis

## Abstract

In this paper, the synthesis, characterization, and biological evaluation of the novel tetrahydropyrimidines—THPMs are described. THPMs are well-known for wide pharmacological activities such as antimicrobial, anticancer, antiviral, etc. This research includes obtained results of in vitro antimicrobial, anticancer, and α-glucosidase inhibitory activities of the eleven novel THPMs. An antibiotic assessment was done against five bacteria (two Gram-positive and three Gram-negative) and five fungi by determining the minimal inhibitory concentration (MIC), using the broth tube dilution method. The most active antibacterial compounds were **4a**, **4b**, and **4d**, while the best antifungal activity was shown by **4e**, **4f**, and **4k**. The lowest MIC value (0.20 mg/mL) was measured for **4e**, **4f**, and **4k** against the *Trichophyton mentagrophytes*. Moreover, examining the α-glucosidase inhibitory activity revealed the compound **4g** as the one with the best activity. The cytotoxic activity was performed on the tumor cell lines (HeLa, K562, and MDA-MB-231) and normal cells (MRC-5). The best antitumor activity was shown by compounds **4b** and **4k** against HeLa cell lines. The influence on cell cycle and mechanism of action of the most active compounds were examined too. Compound **4b** had good antibacterial and anticancer activities, while **4k** showed promising antifungal and anticancer activities.

## 1. Introduction

Pietro Giacomo Biginelli (1860–1937) was an Italian chemist who discovered a one-pot multicomponent reaction. He invented the procedure that would subsequently be named Biginelli synthesis in his honor [1,2]. Despite being the first multicomponent reaction, the scientific community dismisses the Biginelli reaction as outmoded. Nevertheless, since the finding of monastrol as a kinesin-5 inhibitor [3], as well as its anticancer activity, Biginelli chemistry has expanded [4,5,6]. Huge numbers of tetrahydropyrimidines–THPMs (former name 3,4-dihydropyrimidine-2(1*H*)-(thi)ones-DHPMs) have been synthesized using the Biginelli reaction to date. THPMs derivatives have a wide range of biological effects, including anti-inflammatory, antiviral, anticancer, calcium channel inhibition, antibacterial, antifungal, and antioxidant [7,8]. Significant anti-inflammatory activity of the Biginelli thioxo-hybrids was denoted towards various inflammatory diseases [9,10,11]. Although compounds delivered from a Biginelli reaction could possess broad spectra of antiviral activities, among them the most important were those that had inhibitory potential against HIV [12,13,14]. However, it was shown that thio-Biginelli adducts are significantly better calcium channel inhibitors compared to appropriate oxo- and aza-hybrids. In that sense, scientists have found that the (*R*)- was more potent than the (*S*)-enantiomer, which implies that the configuration at the C4 stereocenter plays a key role in this kind of activity [15,16]. Some Biginelli compounds bearing a pyrazole scaffold exhibited good-to-high potency against Mycobacterium tuberculosis [17,18]. In addition, the presence of 1,3-dihydro-2*H*-indol-2-one core in the structure of oxo- and thioxo-Biginelli’s analogs also displayed good antibacterial activities against *Bacillus subtilis*, *E. coli*, and *S. aureus* [19]. Indeed, the most useful aza-nucleophile in Biginelli’s chemistry is urea or *N*-methylurea. However, over the years were shown as quite hard to apply thiourea or thiourea-based nucleophiles in Biginelli’s chemistry [20,21,22,23,24,25]. This fact lies predominantly in its behavior and huge sensitivity towards strong acids and bases, even the Lewis acid/base salt type. In the above-mentioned conditions with heating, it is very hard to apply thioureas in the Biginelli reaction with success. Furthermore, efficient synthesis of Biginelli’s hybrids containing thiourea-based nucleophiles was and is quite challenging. Considering this, our goal was to synthesize a small library of novel THPMs derivatives containing *N*-methylthiourea functionality. Specific objectives were an investigation of their potential for dual biological activities against various microbes and cancer cell lines.

## 2. Materials and Methods

### 2.1. Chemistry

All solvents and substrates (acetyl, benzoyl, and *p*-toluoyl chloride, methyl acetoacetate, *N*-methylthiourea, vanillin, 5-bromovanillin, 5-iodovanillin, 5-nitrovanillin) were purchased from Sigma. Vanillic derivatives **1d–k** were prepared following the procedures described previously in the literature [26]. We determined melting points (Mp) on a Mel-Temp apparatus. For recording IR spectra, we used a Perkin–Elmer Spectrum One FT-IR spectrometer on KBr pellet. A Varian Gemini 200 MHz NMR spectrometer (^1^H at 200 and ^13^C at 50 MHz) was utilized for NMR characterization of compounds **4a–k.** The compounds were dissolved in DMSO-*d_6_*. ^1^H and ^13^C NMR spectra are presented in Supplementary Materials (see Figures S1–S22).

#### Experimental Procedure for Synthesis of **4a**–**k**

A total of 6 mmol of *N*-methylthiourea and 2 mmol of aldehyde were dissolved in the mixture of dioxane/CHCl_3_ (4/1, *v*/*v*) in a 25 mL round-bottom flask. Ten minutes later, 3 drops of HCl (36.5%) were added at room temperature. In all cases, yellow color appeared. Then, two hours later, 6 mmol of methyl acetoacetate and 10 mol% of 2-amino-1-(4-nitrophenyl)-1,3-propanediol (ANP) were loaded. After one day, the solvent was evaporated. Crude solid had been dissolved in ethanol/water mixture (2/1, *v*/*v*). The product was then put in the fridge; after 24 h in those conditions, a precipitate appeared. The formed powder was filtered, and washed with acetone and water. Recrystallization from acetone or ethanol/water was done to reach the desired product with a good purity grade (>95%).

### 2.2. Sample Preparation for MS Analysis

Having in mind that the experiment was not quantitative in nature, care was taken to introduce about 10 ng of the compound into the MS system; a quantity that will provide adequate response without saturating the detector of the instrument. Solid samples were weighted on an analytical balance into the 10 mL volumetric flask up to *cca*. 10 mg and dissolved in HPLC-grade acetonitrile, solutions were vigorously shaken and subsequently sonicated for 10 min at 40 °C in order to dissolve completely. Further dilution to the final concentration of *cca.* 10 ng/uL (in acetonitrile) was prepared directly into the autosampler vials.

Confirmation of theoretical masses was carried out on a Shimadzu (Kyoto, Japan) LC-MS/MS system consisting of two LC-40D × 3 pumps, DGU-405 degassing unit, CTO-40S column oven, SIL-40C × 3 autosampler, and LCMS-8050cl mass spectrometer with ESI interface. The flow injection analysis (FIA) technique was used for direct introduction of the sample into the inlet system avoiding chromatographic separation. Stainless steel tubing (4 m × 0.1 mm ID) was attached instead of the column to the LC pumps/autosampler and mass spectrometer providing a retention time of about 25 s. The mobile phase was 0.1% formic acid in acetonitrile, flow rate was set to 0.2 mL/min.

Positive ESI was used for all compounds with the following parameters: nebulizing gas flow 3 L/min; drying gas flow 10 L/min; heating gas flow10 L/min; interface (capillary) voltage 4.5 kV; interface temperature 350 °C, desolvation temperature 600 °C; desolvation line temperature 250 °C; heat block temperature 300 °C.

The mass spectrometer operated in Q3 scan mode (unit resolution), the scanning range was set from 200 to 800, the scanning speed was 600 u/s.

All materials and methods used for biological examination (antibacterial, antifungal, anticancer, and α-glucosidase inhibitory activities) and crystal structure determination are presented in the Supplementary Materials, File S1.

## 3. Results and Discussion

### 3.1. Chemistry

**Scheme 1:**
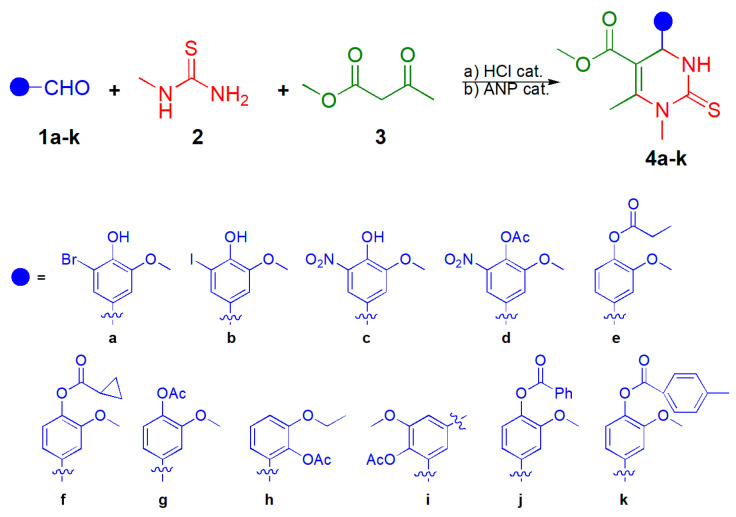
Substrate scope in the synthesis of tetrahydropyrimidines **4a–k**. Reaction conditions: (a) aldehyde and *N*-methylthiourea, dioxane/CHCl_3_, room temperature, (b) methyl acetoacetate, HCl, 2-amino-1-(4-nitrophenyl)-1,3-propanediol (ANP), room temperature.

At the beginning of our research, we were seeking the best reaction conditions to synthesize tetrahydropyrimidine **4a** under a *one*-*pot* manner by applying various acid-based catalysts. For this part of the investigation, methanol, *n*-butanol, and dioxane were utilized as solvents. Under classical Biginelli conditions with hydrochloric acid as a catalyst, the desired product was isolated in a lower yield than expected in all applied solvents. When amidosulfonic acid was used as a catalyst, **4a** was obtained in the yield of 35–50% in the mentioned solvents under reflux. Among the applied solvents, the reaction in dioxane gave the best yield of **4a**. However, reactants **1a**, **2**, and **3** were not fully converted into the expected product. At room temperature, the yield was lower. We decided to apply another strategy preparing an imine intermediate that would allow better production of **4a**. First, we mixed the aldehyde with *N*-methylthiourea, and 3 drops of 36.5% HCl at room temperature in the mixture of dioxane and CHCl_3_. Based on TLC, one hour later, we added active methylene **3**, and substituted 2-amino-1,3-propanediol. The targeted product **4a** was obtained in 63% yield. The reaction without 2-amino-1,3-propanediol only produced imine intermediate. Going forward, different substituted aldehydes were employed to broaden the substrate scope (Scheme 1). The usage of thioureas and their derivatives is quite challenging in Biginelli chemistry, and there are a couple of described procedures in the literature [27,28]; in this research, we performed a reaction at room temperature in a specific mixture of solvents with good yields.

The structures of the isolated Biginelli hybrids (**4a**–**k**) were confirmed by IR, NMR, and ESI-MS spectroscopy. The compound **4j** was suitable for crystallographic analysis. Benzylic proton appeared as a doublet in the range 5.10–5.40 ppm. Thioamide proton and carbon were detected at around 10 ppm, and 178 ppm, respectively. In the ESI-MS spectrum of **4a**, a molecular ion was found at 401 [M]^+^ and 403 [M + 2]^+^ *m*/*z*.

### 3.2. X-ray Crystallography

**Figure 1:**
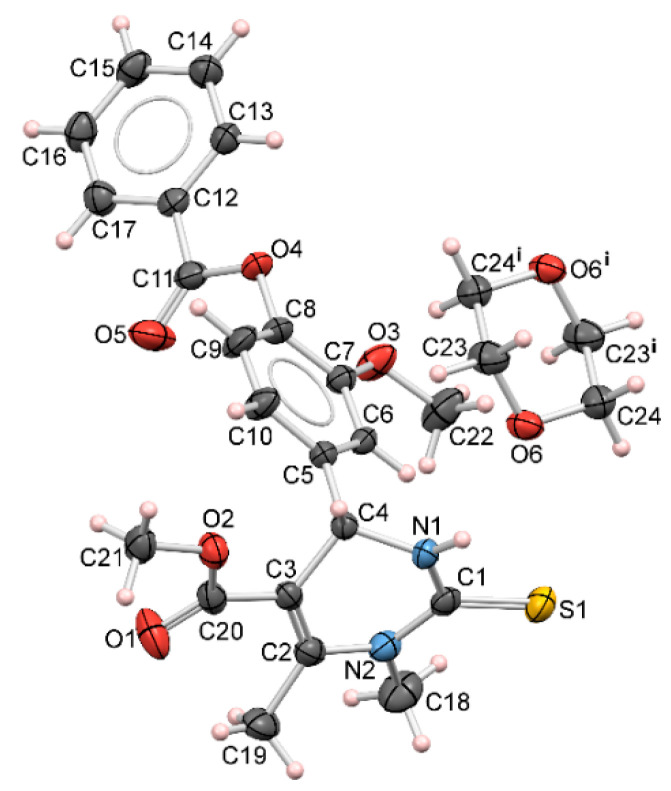
MERCURY [29] drawing of the molecular structure of compound **4j** with labeled non-H atoms. Displacement ellipsoids are shown at 30% probability. Symmetry code: (i) *−x*, *−y+1*, *−z+2*.

Figure 1 shows a perspective view of the molecular structure of methyl 4-(4′-benzoyloxy-3′-methoxyphenyl)-1,2,3,4-tetrahydro-1,6-dimethyl-2-thioxopyrimidine-5-carboxylate (**4j**). A list of selected torsion angles, bond angles, and bond lengths is tabulated in Table 1.

The asymmetric unit of compound **4j** consists of two moieties: one methyl 4-(4′-benzoyloxy-3′-methoxyphenyl)-1,2,3,4-tetrahydro-1,6-dimethyl-2-thioxopyrimidine-5-carboxylate molecule and half of 1,4-dioxane molecule. The conformation of **4j** is best defined by the torsion angles ∠C11—O4—C8—C7 = 103.7 (2)°, ∠C11—O4—C8—C9 = −80.8 (3)°, ∠C10—C5—C4—C3 = 81.9 (2)°, and ∠C6—C5—C4—C3 = −97.22 (18)° (Table 1). Two phenyl moieties are in the nearly gauche position, with the angle between the mean planes of both phenyl rings of 69.63 (10)°. On the other hand, both phenyl rings are nearly perpendicular with the pyrimidine moiety, whereas the angle between the least-squares planes through N1/C1/N2 and C5/C6/C7/C8/C9/C10 atoms is 88.67 (19)°, and through N1/C1/N2 and C12/C13/C14/C15/C16/C17 atoms, it is 82.8 (2)°.

**Figure 2:**
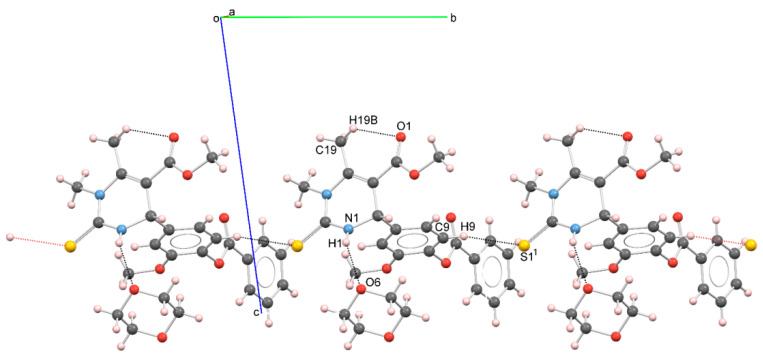
MERCURY [29] drawing showing crystal packing of 4-(4′-benzoyloxy-3′-methoxyphenyl)- 1,2,3,4-tetrahydro-1,6-dimethyl-2-thioxopyrimidine-5-carboxylate (**4j**) viewed along the *a* axis. Intramolecular N—H•••O and C—H•••O contacts are shown as dashed lines. Intermolecular C9—H9•••S1^1*(x, y+1, z)*^ contacts (dashed line) connect molecules in a head-to-tail manner along the *b* axis.

Conformation of molecular structure of **4j** is stabilized by N—H•••O hydrogen bond and C—H•••O contacts (Table 2 and Figure 2).

**Figure 3:**
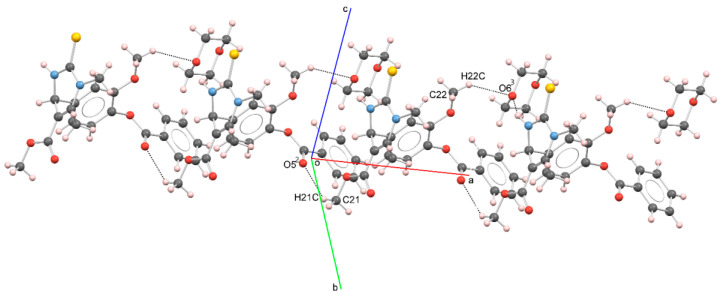
MERCURY [29] drawing of crystal packing of 4-(4′-benzoyloxy-3′-methoxyphenyl)- 1,2,3,4-tetrahydro-1,6-dimethyl-2-thioxopyrimidine-5-carboxylate (**4j**) showing intermolecular C—H•••O contacts (dashed line) connecting molecules in a head-to-tail manner along the *a* axis. Symmetry codes: ^2^: *x−1, y, z*; ^3^: *x+1, y, z*.

Molecular arrangement in the crystal packing of **4j** is governed by a network of C—H•••S and C—H•••O contacts (Table 2) in a head-to-tail manner, whereas C—H•••S form a path of interactions in the [010] direction, forming a thread, as seen in Figure 2. Moreover, the molecules are linked by weak C—H•••O interactions with C21 and C22 methyl atoms as donors and ester atom O5 (*x−1, y, z*) and dioxane atom O6 (*x+1, y, z*) as acceptors in these linkages in the [100] direction (Figure 3).

### 3.3. Biology

#### 3.3.1. Antimicrobial Activity

The obtained MICs of the studied compounds are summarized in Table 3 and Table 4. As shown in the tables, the tested substances manifested relatively strong antimicrobial efficiency. They inhibited growth of all the used microorganisms, except **4e** which acted selectively (this compound had no inhibitory effect on the *Proteus mirabilis*). The measured MICs for the tested components against the used bacteria and fungi ranged from 0.20 to 3.25 mg/mL. The maximum antimicrobial activity showed **4e**, **4f**, and **4k** against the *Trichophyton mentagrophytes* (MIC value was 0.20 mg/mL). The strongest antibacterial effect was found in **4b** and **4d**, while **4f** and **4k** demonstrated the best antifungal effect. The most susceptible microorganism towards all components was *Trichophyton mentagrophytes*.

#### 3.3.2. Cytotoxic Activity of the Compounds

The cytotoxic activity of eleven novel compounds was examined against HeLa, K562, MDA-MB-231, and MRC-5. The tested compounds exerted concentration-dependent cytotoxic effects on cell lines. Among the tested compounds, **4k** and **4b** showed the strongest cytotoxicity against HeLa cells with IC_50_ values of 43.63 µM and 52.59 µM, as presented in Table 5. Compounds **4j** and **4a** exerted lower cytotoxic effects on HeLa cells with IC_50_ values of 78.11 µM and 97.40 µM when compared with the cytotoxicity of the two most active compounds. Compared to compound **4j, 4k** has a methyl group in *para* position of benzoyl fragment; so, probably this structural difference is responsible for better activity and selectivity of compound **4k** on HeLa (43.63 ± 1.49 µM) and K562 (39.11 ± 2.90 µM). In addition, the difference between **4a** and **4b** in position C5′ (**4a** has bromine, and **4b** iodine) provides significantly stronger activity of **4b** on HeLa cell lines. The compounds **4e**, **4f**, **4g**, **4h**, and **4i** exhibited cytotoxic activity against HeLa cells with IC_50_ values ranging from 120.85 µM-197.49 µM, while compounds **4c** and **4d** showed very low cytotoxicity at concentrations up to 200 µM. K562 cells were the most sensitive to the cytotoxic activity of compound **4k** with an IC_50_ value of 39.11 µM.

The four tested compounds **4a**, **4b**, **4h** and **4j** showed moderate cytotoxic activity against K562 cells (IC_50_ values in the range from 67.97 µM–79.94 µM). The observed intensities of cytotoxic activity against K562 cells for the other six examined compounds were in the range from 99.36 µM to 180.21 µM, presented as IC_50_ values. Compound **4k** was the most active against MDA-MB-231 cells (IC_50_ value of 74.12 µM). Cytotoxicity of compounds **4a**, **4b**, **4f**, **4h**, and **4j** on MDA-MB-231 cells was weaker in comparison with activity of **4k** (determined IC_50_ values were in the range from 114.02 µM-161.29 µM). Compounds **4c**, **4d**, **4e**, **4g**, and **4i** had very low cytotoxic activity, with IC_50_ values higher than 200 µM. Each of the eleven examined compounds showed cytotoxic effects on normal fibroblasts MRC-5 (determined IC_50_ values ranging from 77.82 µM–196.08 µM).

Regarding selectivity in the cytotoxic activity, compounds **4a**, **4b**, **4j**, and **4k** exerted higher intensity of cytotoxic activity against HeLa and K562 cells when compared with activity against normal MRC-5 cells. In addition, compound **4a** showed higher cytotoxicity on MDA-MB-231 cells in comparison with cytotoxicity on MRC-5 cells. It is noteworthy that compound **4k** exerted the strongest cytotoxic activity against all tested cancer cell lines. This compound showed good selectivity in the cytotoxic action with selectivity coefficients 2 and 2.23 for HeLa and K562 cells, respectively. The selectivity in the cytotoxic activity against HeLa cells, when compared with activity against MRC-5 cells, was observed for compound **4b** (selectivity coefficient 2.13). Compounds **4k** and **4b**, which showed the strongest selective cytotoxic activity against HeLa cells, were chosen for further analysis of mechanisms of cytotoxic activity.

#### 3.3.3. Effects of the Compounds **4b** and **4k** on Cell Cycle

**Figure 4:**
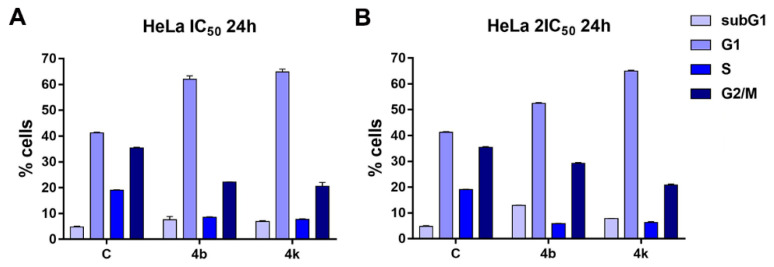
Cell-cycle phase distribution of control HeLa cells and HeLa cells incubated with IC_50_ (**A**) and 2 IC_50_ (**B**) concentrations of compounds **4b** and **4k** during 24 h. The data shown represent the average ± standard deviation of two independent experiments.

The treatment of HeLa cells for 24 h with IC_50_ and 2IC_50_ concentrations of compounds **4b** and **4k** induced an increase in the percentage of dead cells within subG1 phase of the cell cycle when compared with that percentage in the control, untreated cell sample, as it can be seen in Figure 4. In addition, both tested compounds applied at IC_50_ and 2IC_50_ concentrations triggered a pronounced increase in the percentage of HeLa cells in the G1 phase. The observed changes in the amounts of treated cells within subG1 and G1 phases of the cell cycle were accompanied with decreased percentages of cells within the S and G2/M phases. The effect on the cell-cycle phase distribution in HeLa cell samples was concentration-dependent for compound **4b**, while compound **4k** showed similar effects on HeLa cells at both tested concentrations. These results indicate that cytotoxic effects of compounds **4b** and **4k** against HeLa cells could be attributed to an increase in the percentage of cells in the subG1 phase and G1 cell cycle arrest.

#### 3.3.4. Morphological Evaluation of HeLa Cell Death Mode Induced by the Compounds **4b** and **4k**

**Figure 5:**
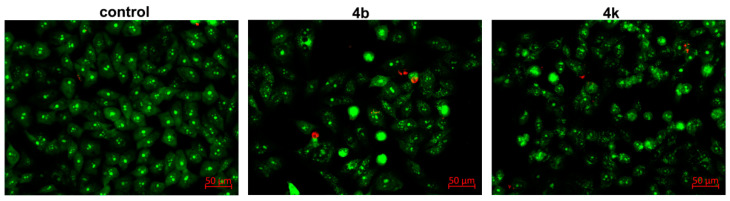
Photomicrographs of acridine orange/ethidium bromide-stained control HeLa cells and HeLa cells incubated with 2IC_50_ concentrations of compounds **4b** and **4k** during 24 h (magnification 20×). Representative photomicrographs are shown.

The photomicrographs of control HeLa cells and HeLa cells incubated for 24 h with 2IC_50_ concentrations of the compounds **4b** and **4k** and stained with a mixture of acridine orange/ethidium bromide are presented in Figure 5. Green-stained rounded cells with condensed chromatin and shrunk nuclei were observed in HeLa cell samples treated with compound **4b**, pointing to the ability of this compound to induce apoptotic cell death. Orange-red-stained shrunken cells with condensed chromatin in later stages of apoptosis were detected as well. Compound **4k** also showed the ability to activate apoptosis in HeLa cells after 24 h treatment, as demonstrated by rounded green cells with condensed nuclei. The proapoptotic effect of compound **4b** was to some extent stronger when comparing with effect of compound **4k**. This result is in accordance with the higher amount of subG1 cells detected in the HeLa cell sample exposed to 2IC_50_ concentration of compound **4b** in comparison with the amount of subG1 cells in cells exposed to compound **4k**.

#### 3.3.5. Inhibitory Effects of Compounds on α-Glucosidase Enzymatic Activity

The tested compounds showed the ability to inhibit α-glucosidase enzymatic activity, with the exception of compounds **4c** and **4j**, as can be seen in Table 6. The obtained IC_50_ values were in the range from 191.80 µM to 1121.91 µM. Among the tested compounds, the compound **4g** exerted the best α-glucosidase inhibitory activity with an IC_50_ value of 191.80 µM. Its activity was stronger than the activity of the anti-diabetic drug acarbose with an IC_50_ value of 304.21 µM. The compounds **4a** and **4b** showed inhibitory activities similar to acarbose. The inhibitory effects of the other examined compounds on α-glucosidase enzymatic activity were weaker when compared with the effect of acarbose.

## 4. Conclusions

Eleven new THPM derivatives were synthesized under mild conditions, while good yields were obtained. For the reaction, methyl acetoacetate, vanillic aldehydes, and *N*-methylthiourea were used. The obtained compounds were characterized by IR, NMR, ES-MS, while molecular structure of compound **4j** has been determined by single-crystal X-ray diffraction analysis. Moreover, their biological activities—antimicrobial, anticancer, and α-glucosidase inhibitory potential—were investigated. The antimicrobial activities of the tested compounds were evaluated against five strains of bacteria and fungi. Compounds **4a**, **4b**, and **4d** have the most effect on bacteria, while the compounds **4e**, **4f**, and **4k** showed the best antifungal activity. Generally, all tested compounds have very good antimicrobial activities which make them worthy of potential pharmacological usage.

The cytotoxic activity was examined against HeLa, K562, MDA-MB-231 cells, and normal human lung fibroblasts MRC-5. The compounds **4k** and **4b** presented the strongest cytotoxicity against HeLa cells with IC_50_ values of 43.63 µM and 52.59 µM. In addition, the effects of the **4k** and **4b** on the cell cycle were examined. Cytotoxic effects of **4k** and **4b** against HeLa cells could be attributed to an increase in the percentage of cells in the subG1 phase and G1 cell cycle arrest. Furthermore, the investigation of the α-glucosidase inhibitory activity revealed compound **4g** as the one with the best activity among all the tested ones. It possesses stronger α-glucosidase inhibitory activity than the anti-diabetic drug acarbose. Further, compounds **4a**, **4b**, and **4i** showed similar activity as acarbose.

Compound **4b** had good antibacterial and anticancer activities, compound **4k** showed promising antifungal and anticancer activities, while compound **4a** has very good α-glucosidase inhibitory activity and antibacterial activity, and compound **4b** possesses similar activity as acarbose, and has good anticancer activity. Therefore, we confirmed the potential of some THPMs to be effective agents for more diseases at the same time. All the results have shown the importance of this type of compound as one with the most diverse, and interesting biological activities. The mentioned facts make THPMs excellent candidates for further investigations.

## Figures and Tables

**Table 1:** Selected geometrical parameters for methyl 4-(4′-benzoyloxy-3′-methoxyphenyl)- 1,2,3,4-tetrahydro-1,6-dimethyl-2-thioxopyrimidine-5-carboxylate (**4j**).

Bond Lengths [Å]			
O1—C20	1.203 (3)	N1—C1	1.339 (2)
O2—C20	1.334 (3)	N1—C4	1.453 (2)
O2—C21	1.442 (2)	N2—C1	1.373 (3)
O3—C7	1.354 (2)	N2—C2	1.410 (3)
O3—C22	1.424 (3)	N2—C18	1.472 (3)
O4—C11	1.354 (2)	C3—C2	1.336 (3)
O4—C8	1.402 (2)	C4—C3	1.517 (2)
O5—C11	1.196 (3)	S1—C1	1.6733 (19)
O6—C24	1.423 (3)	O6—C23	1.425 (3)
**Bond angles [°]**			
C11—O4—C8	116.71 (15)	C1—N1—C4	122.60 (14)
C24—O6—C23	110.08 (16)	N1—C1—N2	115.90 (16)
C20—O2—C21	116.98 (17)	N1—C1—S1	120.25 (14)
**Torsion angles [°]**			
C4—N1—C1—N2	−23.7 (2)	C2—C3—C20—O1	−19.9 (3)
N1—C4—C3—C2	−31.1 (2)	C4—C3—C20—O1	157.6 (2)
C2—N2—C1—N1	−10.4 (2)	C2—C3—C20—O2	162.28 (16)
N1—C4—C3—C20	151.29 (15)	C4—C3—C20—O2	−20.2 (2)
C18—N2—C1—S1	−8.3 (3)	O3—C7—C8—O4	−3.0 (3)
C2—N2—C1—S1	20.2 (3)	C11—O4—C8—C9	−80.8 (3)
C1—N2—C2—C3	169.09 (13)	C11—O4—C8—C7	103.7 (2)
C5—C4—C3—C2	92.44 (18)	C17—C12—C11—O5	9.3 (3)

**Table 2:** Intramolecular and intermolecular contact parameters in the crystal structure of methyl 4-(4′-benzoyloxy-3′-methoxyphenyl)-1,2,3,4-tetrahydro-1,6-dimethyl-2-thioxopyrimidine-5-carboxylate (**4j**).

*D*—H•••A	*D*—H	H•••A	*D*•••A	*D*—H•••A
N1—H1•••O6	0.86	2.23	2.897 (2)	134.9
C19—H19B•••O1	0.93	2.95	3.809 (2)	154.0
C9—H9•••S1 ^1^	0.96	2.61	3.363 (3)	135.7
C21—H21C•••O5 ^2^	0.96	2.61	3.363 (3)	135.7
C22—H22C•••O6 ^3^	0.96	2.62	3.407 (3)	139.5

Symmetry codes: ^1^ *x*, *y* + 1, *z*; ^2^ *x* − 1, *y*, *z*; ^3^ *x* + 1, *y*, *z*.

**Table 3:** The antibacterial activity of the tested compounds.

Tested Compounds	*Staphylococcus aureus*	*Bacillus subtilis*	*Klebsiella oxytoca*	*Proteus* *mirabilis*	*Escherichia coli*
	MIC (mg/mL)				
**4a**	1.62	3.25	1.62	0.81	1.62
**4b**	1.62	3.25	1.62	0.81	1.62
**4c**	1.62	3.25	1.62	1.62	1.62
**4d**	0.81	3.25	1.62	0.81	1.62
**4e**	3.25	3.25	3.25	ND	1.62
**4f**	3.25	3.25	3.25	1.62	3.25
**4g**	3.25	3.25	1.62	0.81	1.62
**4h**	3.25	3.25	3.25	3.25	3.25
**4i**	3.25	3.25	1.62	1.62	1.62
**4j**	3.25	3.25	3.25	1.62	3.25
**4k**	3.25	3.25	3.25	3.25	3.25
Streptomycin	0.031	0.016	0.008	0.062	0.062

ND—not detected.

**Table 4:** The antifungal activity of the tested compounds.

Tested Compounds	*Trichophyton* *mentagrophytes*	*Mucor mucedo*	*Penicillium italicum*	*Aspergillus flavus*	*Aspergillus niger*
	MIC (mg/mL)				
**4a**	0.40	1.62	1.62	1.62	1.62
**4b**	0.40	1.62	1.62	1.62	0.81
**4c**	0.40	1.62	1.62	0.40	0.81
**4d**	0.40	1.62	1.62	1.62	1.62
**4e**	0.20	1.62	1.62	1.62	1.62
**4f**	0.20	1.62	1.62	1.62	0.81
**4g**	0.40	1.62	1.62	1.62	1.62
**4h**	0.81	1.62	1.62	1.62	1.62
**4i**	0.81	1.62	1.62	1.62	1.62
**4j**	0.40	1.62	1.62	1.62	1.62
**4k**	0.20	1.62	1.62	0.81	1.62
Fluconazole	0.25	1	1	1	0.5

**Table 5:** Cytotoxic activity of compounds.

Tested Compounds	HeLa	K562	MDA-MB-231	MRC-5
	IC_50_ [µM] Average ± SD			
**4a**	97.40 ± 5.40	78.98 ± 7.49	144.50 ± 8.29	186.92 ± 4.66
**4b**	52.59 ± 4.45	76.83 ± 5.01	115.65 ± 8.71	111.87 ± 10.85
**4c**	>200	180.21 ± 9.15	>200	195.83 ± 5.89
**4d**	>200	164.66 ± 8.50	>200	192.71 ± 10.32
**4e**	197.22 ± 3.93	149.08 ± 5.74	>200	127.98 ± 3.20
**4f**	135.34 ± 9.32	122.91 ± 8.52	161.29 ± 9.69	196.08 ± 5.54
**4g**	197.49 ± 3.55	152.68 ± 2.58	>200	193.22 ± 9.59
**4h**	120.85 ± 9.96	79.94 ± 6.73	114.02 ± 12.86	77.82 ± 2.57
**4i**	169.06 ± 4.12	99.36 ± 9.38	>200	152.34 ± 10.15
**4j**	78.11 ± 5.78	67.97 ± 6.53	122.61 ± 2.19	104.17 ± 8.61
**4k**	43.63 ± 1.49	39.11 ± 2.90	74.12 ± 1.25	87.23 ± 7.31
*cis*Pt	4.91 ± 0.74	6.89 ± 0.21	14.74 ± 0.36	9.35 ± 1.29

The results are presented as average ± standard deviation of three independent experiments performed in triplicate.

**Table 6:** α-glucosidase inhibitory activity of the compounds.

Compounds	IC_50_ [µM]
**4a**	312.91 ± 7.73
**4b**	291.77 ± 6.67
**4c**	ND
**4d**	1121.91 ± 20.59
**4e**	674.81 ± 5.65
**4f**	767.91 ± 5.91
**4g**	191.80 ± 5.95
**4h**	932.75 ± 25.16
**4i**	418.02 ± 5.68
**4j**	ND
**4k**	1083.41 ± 54.54
acarbose	304.21 ± 14.62

The results are presented as average ± standard deviation of two experiments. ND—non-determined

## Data Availability

Not applicable.

## Supplementary Materials

The following supporting information can be downloaded at: https://www.mdpi.com/article/10.3390/pharmaceutics14102254/s1, Supplementary Materials File S1: Materials and methods for biological examination and crystal structure determination; Figures S1–S22: NMR spectra; Table S1: Crystallographic data and refinement parameters for **4j**; Supplementary Materials File S2: MS spectra of **4a**–**k**. Refs. [30,31,32,33,34,35,36,37,38,39,40] are cited in Supplementary Materials File S1.
