# Supplementary material for: Synthesis, Characterization, and Biological Evaluation of Tetrahydropyrimidines: Dual-Activity and Mechanism of Action
Source: Pharmaceutics. 2022 Oct 21;14(10):2254. doi: 10.3390/pharmaceutics14102254 (PMC9610702; doi:10.3390/pharmaceutics14102254)
Supplement: Supplementary file 1 [file pharmaceutics-14-02254-s001.zip › Supplementary Materials_File 1_revised.pdf]

PHARMACEUTICS

Supplementary Materials associated with the paper

Synthesis, characterization, and biological evaluation of tetrahydropyrimidines: Dual-activity and mechanism of action

Emilija Milović,[1] Nenad Janković,[1*] Jelena Petronijević,[2] Nenad Joksimović,[2] Marijana Kosanić,[3] Tatjana Stanojković,[4] Ivana Matić,[4] Nađa Grozdanić,[4] Olivera Klisurić,[5] and Srđan Stefanović[6]

[1]University of Kragujevac, Institute for Information Technologies Kragujevac, Department of Sciences, Jovana Cvijića bb, 34000 Kragujevac, Serbia.

[2]University of Kragujevac, Faculty of Science, Department of Chemistry, Radoja Domanovića 12, 34000 Kragujevac, Serbia.

[3]University of Kragujevac, Faculty of Science, Department of Biology and Ecology, Radoja Domanovića 12, 34000 Kragujevac, Serbia.

[4]Institute of Oncology and Radiology of Serbia, Pasterova 14, 11000, Belgrade, Serbia

[5] University of Novi Sad, Faculty of Science, Department of Physics, Trg Dositeja Obradovića 3, 21000 Novi Sad, Serbia

[6]Institute of Meat Hygiene and Technology, Kaćanskog 13, 11000 Belgrade, Serbia

Corresponding author's e-mail address:nenad.jankovic@kg.ac.rs

Contents

Materials and methods for biological examinations

Crystal structure determination

Experimental data of **4a-k**

NMR spectra of **4a-k**

^{1}H NMR of methyl 4-(3’-bromo-4’-hydroxy-5’-methoxyphenyl)-1,2,3,4-tetrahydro-1,6-dimethyl-2-thioxopyrimidine-5-carboxylate **4a**

^{13}C NMR of methyl 4-(3’-bromo-4’-hydroxy-5’-methoxyphenyl)-1,2,3,4-tetrahydro-1,6-dimethyl-2-thioxopyrimidine-5-carboxylate **4a**

^{1}H NMR of methyl 1,2,3,4-tetrahydro-4-(4’-hydroxy-3’-iodo-5’-methoxyphenyl)-1,6-dimethyl-2-thioxopyrimidine-5-carboxylate **4b**

^{13}C NMR of methyl 1,2,3,4-tetrahydro-4-(4’-hydroxy-3’-iodo-5’-methoxyphenyl)-1,6-dimethyl-2-thioxopyrimidine-5-carboxylate **4b**

^{1}H NMR of methyl 1,2,3,4-tetrahydro-4-(4’-hydroxy-3’-methoxy-5’-nitrophenyl)-1,6-dimethyl-2-thioxopyrimidine-5-carboxylate **4c**

^{13}C NMR of methyl 1,2,3,4-tetrahydro-4-(4’-hydroxy-3’-methoxy-5’-nitrophenyl)-1,6-dimethyl-2-thioxopyrimidine-5-carboxylate **4c**

^{1}H NMR of methyl 4-(4’-acetoxy-3’-methoxy-5’-nitrophenyl)-1,2,3,4-tetrahydro-1,6-dimethyl-2-thioxopyrimidine-5-carboxylate **4d**

^{13}C NMR of methyl 4-(4’-acetoxy-3’-methoxy-5’-nitrophenyl)-1,2,3,4-tetrahydro-1,6-dimethyl-2-thioxopyrimidine-5-carboxylate **4d**

^{1}H NMR of methyl 1,2,3,4-tetrahydro-4-(3’-methoxy-4’-propionyloxyphenyl)-1,6-dimethyl-2-thioxopyrimidine-5-carboxylate **4e**

^{13}C NMR of methyl 1,2,3,4-tetrahydro-4-(3’-methoxy-4’-propionyloxyphenyl)-1,6-dimethyl-2-thioxopyrimidine-5-carboxylate **4e**

^{1}H NMR of methyl 4-(4’-(cyclopropanecarboxyloyloxy)-3’-methoxyphenyl)-1,2,3,4-tetrahydro-1,6-dimethyl-2-thioxopyrimidine-5-carboxylate **4f**

^{13}C NMR of methyl 4-(4’-(cyclopropanecarboxyloyloxy)-3’-methoxyphenyl)-1,2,3,4-tetrahydro-1,6-dimethyl-2-thioxopyrimidine-5-carboxylate **4f**

^{1}H NMR of methyl 4-(4’-acetoxy-3’-methoxyphenyl)-1,2,3,4-tetrahydro-1,6-dimethyl-2-thioxopyrimidine-5-carboxylate **4g**

^{13}C NMR of methyl 4-(4’-acetoxy-3’-methoxyphenyl)-1,2,3,4-tetrahydro-1,6-dimethyl-2-thioxopyrimidine-5-carboxylate **4g**

^{1}H NMR of methyl 4-(2’-acetoxy-3’-ethoxyphenyl)-1,2,3,4-tetrahydro-1,6-dimethyl-2-thioxopyrimidine-5-carboxylate **4h**

^{13}C NMR of methyl 4-(2’-acetoxy-3’-ethoxyphenyl)-1,2,3,4-tetrahydro-1,6-dimethyl-2-thioxopyrimidine-5-carboxylate **4h**

^{1}H NMR of 4,4'-(1,3-phenylene)bis(5-methyl 1,2,3,4-tetrahydro-1,6-dimethyl-2-thioxopyrimidine-5-carboxylate) **4i**

^{13}C NMR of 4,4'-(1,3-phenylene)bis(5-methyl 1,2,3,4-tetrahydro-1,6-dimethyl-2-thioxopyrimidine-5-carboxylate) **4i**

^{1}H NMR of methyl 4-(4’-benzoyloxy-3’-methoxyphenyl)-1,2,3,4-tetrahydro-1,6-dimethyl-2-thioxopyrimidine-5-carboxylate **4j**

^{13}C NMR of methyl 4-(4’-benzoyloxy-3’-methoxyphenyl)-1,2,3,4-tetrahydro-1,6-dimethyl-2-thioxopyrimidine-5-carboxylate **4j**

^{1}H NMR of methyl 4-(4’-(4’’-methylbenzoyloxy)-3’-methoxyphenyl)-1,2,3,4-tetrahydro-1,6-dimethyl-2-thioxopyrimidine-5-carboxylate **4k**

^{13}C NMR of methyl 4-(4’-(4’’-methylbenzoyloxy)-3’-methoxyphenyl)-1,2,3,4-tetrahydro-1,6-dimethyl-2-thioxopyrimidine-5-carboxylate **4k**

Materials and methods for biological examinations

Antimicrobial activity

Antimicrobial activities of tested compounds were evaluated against five strains of bacteria: *Staphylococcus aureus* (ATCC 25923), *Bacillus subtilis* (ATCC 6633), *Klebsiella oxytoca* (ATCC 8724), *Proteus mirabilis* (ATCC 29906) and *Escherichia coli* (ATCC 25922), and five species of fungi: *Trichophyton mentagrophytes* (ATCC 9533),*Mucor mucedo* (ATCC 20094), *Penicillium italicum* (ATCC 10454), *Aspergillus flavus* (ATCC 9170), and *Aspergillus niger* (ATCC 16888), obtained from the American Type Culture Collection (ATCC).

The bacteria isolates were picked from overnight cultures in Mueller-Hinton agar and suspensions were prepared in sterile distilled water. The turbidity of suspensions was adjusted by comparing with 0.5 McFarland's standard to approximately 108 CFU/mL.

Fungal suspensions were prepared from 3- to 7-day-old cultures that grew on a potato dextrose agar. The spores were rinsed with sterile distilled water, used to determine turbidity spectrophotometrically at 530 nm NCCLS (1998). The resulting suspensions were approximately 106 CFU/mL.

The 96-well microtiter assay using resazurin as the indicator of cell growth [1], was employed for the determination of the minimum inhibitory concentration (MIC) of the active components. Starting solutions of tested compounds were obtained by dissolving it in 5% DMSO. Next, serial twofold dilutions of tested compounds were made in a concentration range from 13 to 0.015 mg/mL in sterile 96-well plates containing Mueller-Hinton broth for bacterial cultures and a SD broth for fungal cultures. After that, diluted bacterial and fungal suspensions were added to appropriate wells and finally, resazurin solution was added as an indicator to each well. The inoculated plates were incubated at 37°C for 24 h for bacteria and 28°C for 72 h for fungi. The MIC was determined visually and defined as the lowest concentration of tested compounds that prevented resazurin color change from blue to pink. Streptomycin was used as a positive control. Solvent control test was performed to study an effect of 5% DMSO on the growth of microorganism.

Determination of cytotoxic activity

Human cervical adenocarcinoma HeLa, human chronic myelogenous leukemia K562, human breast adenocarcinoma MDA-MB-231, and normal human lung fibroblasts MRC-5 cell lines were grown in RPMI-1640 medium with 10% fetal bovine serum, L-glutamine, and penicillin-streptomycin solution, while MDA-MB-231 cells were grown in DMEM medium, supplemented with 10% fetal bovine serum, L-glutamine, and penicillin-streptomycin solution.

HeLa (3000 cells per well), MDA-MB-231 (5000 cells per well), and MRC-5 cells (5000 cells per well) were plated into the 96-well cell culture plates. The cell cultures were incubated at 37 °C in a CO_2 incubator. Adherent cell lines were incubated for 20 h before addition of compounds. K562 cells (5000 cells per well) were seeded into the 96-well plates 2 h before addition of compounds. The cells were treated with tested compounds applied at five different concentrations (double dilutions) ranging from 12.5 µM to 200 µM for 72 h. The complete growth medium was added to control cell samples in each plate. After 72 h incubation, the survival of cells was determined by MTT assay according to the protocol established by Mosmann [2], and modified by Ohno and Abe [3]. The solution of MTT was added to blank and cell samples. The plates were incubated for 4 h at 37 °C, and afterwards, the 10% solution of sodium dodecyl sulfate was added to the wells. The absorbance was measured the next day at 570 nm using Thermo Scientific Multiskan EX plate reader. Three independent experiments performed were performed.

Cell cycle analysis

The effects of the compounds **4b** and **4k** on cell cycle phase distribution of HeLa cells after 24 h treatment were investigated by flow cytometric analysis, as described previously [4]. HeLa cells (200000 cells per well) were plated into the 6-well plates. After 20 h incubation, the medium was replaced with fresh growth medium containing tested compounds at IC_{50} and $2IC_{50}$ concentrations determined after 72 h treatment. The cells were exposed to the compounds for 24 h. Subsequently, the cells were harvested by trypsinization, washed with phosphate buffered saline (PBS), and fixed in 70% cold ethanol. The cell samples were stored at -20 °C for one week before analysis. At the day of analysis, the ethanol was removed after centrifugation and the cell samples were washed with PBS. The cell pellets were resuspended in PBS with RNase A, and incubated for 30 min at 37°C. Afterwards, the propidium iodide solution was added to the cells. Distribution of HeLa cells within specific phases of the cell cycle was determined on the BD FACSCaliburTM flow cytometer using BD CellQuestProTM software (BD Biosciences) for data acquisition and analysis. For each cell sample 10000 events were collected in a gate and analyzed. Two independent experiments were performed.

Morphological analysis of cell death by fluorescence microscopy

The morphological hallmarks of cell death triggered by selected compounds **4b** and **4k** in HeLa cells after 24 h exposure was examined by fluorescence microscopy. HeLa cells were seeded into the 6-well plates on coverslips (100000 cells per well). Twenty hours later, the medium was removed and fresh growth medium with $2IC_{50}$ concentrations of the compounds was added. After 24 h incubation the control and treated HeLa cells on coverslips were stained with a mixture of acridine orange and ethidium bromide nucleic acid dyes and their morphological characteristics were evaluated on Carl Zeiss PALM MicroBeam microscope with Axio Observer.Z1 and AxioCamMRm camera.

α-glucosidase inhibitory activity

The possible α-glucosidase inhibitory effects of the tested compounds were examined according to modified procedure reported by McCue et al. [5], and described previously in more detail [6]. In brief, the solution of α-glucosidase from *Saccharomyces cerevisiae* was prepared at a concentration of 400 mU/mL in a 0.1 M phosphate buffer (pH = 6.8). Mixtures of solutions of compounds in a phosphate buffer and enzyme solution were incubated in 96-well microplates with flat bottom at 37 °C for 15 min. The enzymatic reaction was initiated by adding substrate solution (1.5 mg/mL PNP-G (p-nitrophenyl α-D-glucopyranoside) in the buffer). The first absorbance of the reaction mixture A1 was measured at 405 nm. Afterwards the mixture was incubated at 37 °C for 15 min and the second absorbance A2 was measured at 405 nm at the end of incubation. Acarbose was used as a positive control. Percentage of the α-glucosidase inhibition was determined according to the equation $100 \times (A2S – A1S)/(A2B-A1B)$, where A1B, A2B and A1S, A2S represent the absorbance of the blank (phosphate buffer, DMSO, enzyme dilution, and PNP-G dilution) and the sample, respectively. All experiments were done in duplicate. The concentration of the compound which inhibited 50% of α-glucosidase enzymatic activity was defined as IC_{50}.

Crystal structure determination

X-ray diffraction data for methyl 4-(4’-benzoyloxy-3’-methoxyphenyl)- 1,2,3,4-tetrahydro-1,6-dimethyl-2-thioxopyrimidine-5-carboxylate (**4j**) was collected at room temperature on an Rigaku (Oxford Diffraction) Gemini S diffractometer. For data collection and data integration we used CrysAlisPro and CrysAlis RED software packages [7]. Absorption effects on collected data for **4j** were corrected using the multi-scan method implemented in SCALE3 ABSPACK [7]. Multi-scan method [8] mostly used for area-detectors data collection, models the absorption surface using spherical harmonics which are based on differences between equivalent reflections. Crystal structure of compound 4j was solved and refined using programs SHELXT [9] and SHELXL-2018/3 [10], respectively. MERCURY [11] and OLEX2 [12] software were employed for preparation of material for publication. All non-hydrogen atoms in the crystal structure of compound **4j** were refined ani-sotropically. On the other hand, hydrogen atoms were subjected to constrained isotropic refinement. Table S1 contains all crystalographic and refinement parameters for compound **4j**.
Accession Code CCDC 2169553 (compound **4j**) contain the supplementary crystallographic data for this paper. These data can be obtained free of charge via www.ccdc.cam.ac.uk/data_request/cif, or by emailing data_request@ccdc.cam.ac.uk, or by contacting The Cambridge Crystallographic Data centre, 12 Union Road, Cambridge CB2 1EZ, UK; fax: + 44 1223 336,033.

Table S1. Crystallographic data and refinement parameters for **4j**

Crystal data	
Compound	**4j**
Chemical formula	$C_{24}H_{26}N_2O_6S$
M_r	470.53
Crystal system	Triclinic
Space group	$P\bar{1}$
a (Å)	8.7316 (4)
b (Å)	10.4614 (5)
c (Å)	13.5621 (7)
α (°)	80.546 (4)
β (°)	81.700 (4)
γ (°)	74.121 (4)
V (Å^3)	1168.90 (10)

Z	2
D_x (Mg m^{-3})	1.337
Radiation type	Cu $K\alpha$ (λ = 1.54184 Å)
μ (mm^{-1})	1.59
Crystal size (mm)	0.57 × 0.40 × 0.35
Data collection	
Absorption correction	Multi-Scan
T_{min}, T_{max}	0.755, 1.000
Reflections collected	7855
Independent reflections	4451
Observed reflections [$I > 2\sigma(I)$]	3870
R_{int}	0.021
Range of h, k, l	$h = -10 \rightarrow 10$, $k = -12 \rightarrow 12$, $l = -15 \rightarrow 16$
ϑ values (°)	$\vartheta_{max} = 71.7$, $\vartheta_{min} = 3.3$
Refinement	
$R[F^2 > 2\sigma(F^2)]$, $wR(F^2)$	0.0467, 0.1289
R[*all data*], $wR2$	0.0527, 0.1358
Goodness-of-fit (S)	1.041
No. of reflections	4451
No. of parameters	302
No. of restraints	0
$\Delta\rho_{max}$, $\Delta\rho_{min}$ (e Å^{-3})	0.28, −0.35

CCDC no.	2169553

Experimental data of 4a-k

Abbreviations for the NMR signals are: s = singlet, d = doublet, t = triplet, m = multiplet, and dd = doublet of doublet.

methyl 4-(3'-bromo-4'-hydroxy-5'-methoxyphenyl)-1,2,3,4-tetrahydro-1,6-dimethyl-2-thioxopyrimidine-5-carboxylate 4a

White powder; Yield: 63%; Mp= 208°C; IR (ATR) *v*: 3460, 3203, 1695, 1689, 1085 cm^{-1};^{1}H NMR (200 MHz, DMSO-d_6): δ 9.83 (d, *J* = 4.6 Hz, 1H), 9.55 (s, 1H), 6.77 (s, 1H), 6.81 (s, 1H), 5.12 (d, *J* = 4.5 Hz, 1H), 3.78 (s, 3H), 3.65 (s, 3H), 3.48 (s, 3H), 2.54 (s, 3H) ppm;^{13}C NMR (50 MHz, DMSO-d_6): δ 178.1, 165.7, 148.5, 148.4, 143.4, 133.9, 121.5, 109.3, 109.2, 105.0, 56.2, 51.7, 51.6, 36.3, 16.5 ppm; ESI-MS (m/z): 401 $[M]^+$ and 403 $[M + 2]^+$.

methyl 1,2,3,4-tetrahydro-4-(4'-hydroxy-3'-iodo-5'-methoxyphenyl)-1,6-dimethyl-2-thioxopyrimidine-5-carboxylate 4b

White powder; Yield: 73%; Mp = 198°C; IR (ATR) *v*: 3460, 3211, 1695, 1647, 1085 cm^{-1};^{1}H NMR (200 MHz, DMSO-d_6): δ 9.82 (d, *J* = 4.6 Hz, 1H), 9.60 (s, 1H), 7.02 (d, *J* = 1.9 Hz, 1H), 6.76 (d, *J* = 2.0 Hz, 1H), 5.10 (d, *J* = 4.6 Hz, 1H), 3.76 (s, 3H), 3.65 (s, 3H), 3.48 (s, 3H), 2.53 (s, 3H) ppm; ^{13}C NMR (50 MHz, DMSO-d_6):δ 178.1, 165.8, 148.3, 147.1, 145.9, 134.8, 127.3, 109.9, 105.1, 84.7, 56.1, 51.7, 51.4, 36.3, 16.5 ppm; ESI-MS (m/z): 449 $[M + H]^+$.

methyl 1,2,3,4-tetrahydro-4-(4'-hydroxy-3'-methoxy-5'-nitrophenyl)-1,6-dimethyl-2-thioxopyrimidine-5-carboxylate 4c

Orange powder; Yield: 77%; Mp = 190°C; IR (ATR) *v*: 3325, 1678, 1624, 1544, 1510, 1074 cm^{-1};^{1}H NMR (200 MHz, DMSO-d_6): δ10.52 (s, 1H), 9.91 (d, *J* = 4.7 Hz, 1H), 7.19 (d, *J* = 2.1 Hz, 1H), 7.09 (d, *J* = 2.0 Hz, 1H), 5.19 (d, *J* = 4.6 Hz, 1H), 3.85 (s, 3H), 3.65 (s, 3H), 3.48 (s, 3H), 2.55 (s, 3H) ppm;^{13}C NMR (50 MHz, DMSO-d_6):δ 178.3, 165.6, 149.7, 148.9, 142.2, 136.9, 132.5, 114.1, 112.9, 104.5, 56.7, 51.8, 51.5, 36.4, 16.5 ppm; ESI-MS (m/z): 368 $[M + H]^+$.

methyl 4-(4'-acetoxy-3'-methoxy-5'-nitrophenyl)-1,2,3,4-tetrahydro-1,6-dimethyl-2-thioxopyrimidine-5-carboxylate 4d

Yellow powder; Yield: 46%; Mp =181°C; IR (ATR) *v*: 3178, 3024, 1772, 1708, 1537, 1346 cm^{-1};^{1}H NMR(200 MHz, DMSO-d_6): δ10.01 (d, *J* = 4.7 Hz, 3H), 7.40 (s 1H), 7.33 (s 1H), 5.30 (d, *J* = 4.6 Hz, 1H), 3.86 (s, 3H), 3.67 (s, 3H), 3.49 (s, 3H), 2.57 (s, 3H), 2.32 (s, 3H) ppm;^{13}C NMR (50 MHz, DMSO-d_6):δ 178.4, 167.7, 165.5, 152.5, 149.5, 142.6, 141.3, 132.3, 115.5, 113.2, 103.9, 57.0, 51.9, 51.7, 36.5, 20.3, 16.5 ppm; ESI-MS (m/z): 410 $[M + H]^+$.

methyl 1,2,3,4-tetrahydro-4-(3'-methoxy-4'-propionyloxyphenyl)-1,6-dimethyl-2-thioxopyrimidine-5-carboxylate 4e

White powder; Yield: 28%; Mp = 194°C; IR (ATR) *v*: 3213, 1755, 1712, 1647, 1413 cm^{-1};^{1}H NMR (200 MHz, DMSO-d_6): δ9.91 (d, *J* = 4.7 Hz, 1H), 7.04 (d, *J* = 8.1 Hz, 1H), 6.95 (d, *J* = 1.9 Hz, 1H), 6.74 (dd, *J* = 8.2, 1.9 Hz, 1H), 5.22 (d, *J* = 4.6 Hz, 1H), 3.73 (s, 3H), 3.67 (s, 3H), 3.49 (s, 3H), 2.67 – 2.42 (m, 5H), 1.11 (t, J = 7.5 Hz, 3H) ppm;^{13}C NMR (50 MHz, DMSO-d_6):δ 178.3, 172.0, 165.8, 150.9, 148.4, 140.9, 138.9, 123.0, 117.7, 110.7, 105.2, 55.8, 52.0, 51.8, 36.4, 26.8, 16.5, 9.2 ppm; ESI-MS (m/z): 379 $[M + H]^+$.

methyl 4-(4’-(cyclopropanecarboxyloyloxy)-3’-methoxyphenyl)-1,2,3,4-tetrahydro-1,6-dimethyl-2-thioxopyrimidine-5-carboxylate 4f

White powder; Yield: 75%; Mp = 173°C; IR (ATR) *v*: 3196, 1735, 1708, 1651, 1537, 1138 cm^{-1};^1H NMR (200 MHz, DMSO-d_6); δ9.90 (d, *J* = 4.7 Hz, 1H), 7.04 (d, *J* = 8.1 Hz, 1H), 6.94 (d, *J* = 1.9 Hz, 1H), 6.73 (dd, *J* = 8.2, 1.9 Hz, 1H), 5.21 (d, *J* = 4.6 Hz, 1H), 3.73 (s, 3H), 3.66 (s, 3H), 3.49 (s, 3H), 2.53 (s, 3H), 1.81-1.94 (m, 1H), 0.92 – 1.09 (m, 3H) ppm; ^{13}C NMR (50 MHz, DMSO-d_6);δ 178.3, 172.3, 165.7, 150.9, 148.4, 140.9, 138.7, 123.0, 117.7, 110.6, 105.2, 55.8, 52.0, 51.7, 36.3, 16.5, 12.5, 9.1 ppm; ESI-MS (m/z): 391 $[M + H]^+$and 413 $[M + Na]^+$.

methyl 4-(4’-acetoxy-3’-methoxyphenyl)-1,2,3,4-tetrahydro-1,6-dimethyl-2-thioxopyrimidine-5-carboxylate 4g

White powder; Yield: 66%; Mp = 170°C; IR (ATR) *v*: 3188, 1761, 1707, 1616, 1074 cm^{-1};^1H NMR (200 MHz, DMSO-d_6): δ9.91 (d, *J* = 4.7 Hz, 1H), 7.04 (d, *J* = 8.1 Hz, 1H), 6.95 (d, *J* = 1.9 Hz, 1H), 6.74 (dd, *J* = 8.2, 2.0 Hz, 1H), 5.22 (d, *J* = 4.6 Hz, 1H), 3.74 (s, 3H), 3.66 (s, 3H), 3.49 (s, 3H), 2.53 (s, 3H), 2.24 (s, 3H) ppm;^{13}C NMR (50 MHz, DMSO-d_6):δ 178.3, 168.5, 165.7, 150.8, 148.4, 140.9, 138.8, 123.0, 117.7, 110.6, 105.1, 55.8, 52.0, 51.7, 36.4, 20.5, 16.5 ppm; ESI-MS (m/z): 365 $[M + H]^+$ and 387 $[M + Na]^+$.

methyl 4-(2’-acetoxy-3’-ethoxyphenyl)-1,2,3,4-tetrahydro-1,6-dimethyl-2-thioxopyrimidine-5-carboxylate 4h

White powder; Yield: 79%; Mp = 150°C; IR (ATR) *v*: 3305, 3209, 1768, 1689, 1633 cm^{-1};^1H NMR (200 MHz, DMSO-d_6): δ9.68 (d, *J* = 5.0 Hz, 1H), 7.25 – 7.07 (m, 1H), 7.03 (dd, *J* = 8.2, 1.5 Hz, 1H), 6.74 (dd, *J* = 7.7, 1.5 Hz, 1H), 5.38 (d, *J* = 4.9 Hz, 1H), 4.00 (q, *J* = 6.9 Hz, 2H), 3.56 (s, 3H), 3.50 (s, 3H), 2.51 (s, 3H), 2.29 (s, 3H), 1.27 (t, *J* = 6.9 Hz, 3H);^{13}C NMR (50 MHz, DMSO-d_6):δ 177.9, 168.0, 165.6, 150.3, 147.9, 134.4, 126.4, 118.7, 113.2, 104.5, 64.2, 51.6, 47.8, 36.4, 20.7, 16.4, 14.7 ppm; ESI-MS (m/z): 379 $[M + H]^+$ and 401 $[M + Na]^+$.

4,4'-(1,3-phenylene)bis(5-methyl 1,2,3,4-tetrahydro-1,6-dimethyl-2-thioxopyrimidine-5-carboxylate)4i

White powder; Yield: 25%; Mp =220°C; IR (ATR) *v*:3254, 1760, 1684, 1624 cm^{-1};^1H NMR(200 MHz, DMSO-d_6): δ9.90 (d, *J* = 4.7 Hz, 1H), 9.69 (d, *J* = 5.0 Hz, 1H), 6.91-6.97 (m, 1H), 6.46-6.49 (m, 1H), 5.30 (d, *J* = 4.9 Hz, 1H), 5.18 (d, *J* = 4.6 Hz, 1H), 3.72 (s, 3H), 3.67 (s, 3H), 3.49 (s, 3H), 3.46 (s, 3H), 2.51 (m, 6H), 2.29 (s, 3H);^{13}C NMR (50 MHz, DMSO-d_6):δ 178.4, 178.1, 168.0, 165.7, 165.5, 151.0, 148.3, 147.6, 140.3, 136.1, 134.3, 114.9, 110.1, 105.2, 104.7, 56.0, 51.8, 51.7, 47.6, 36.4, 20.7, 16.7 ppm; ESI-MS (m/z): 563 $[M + H]^+$.

methyl 4-(4’-benzoyloxy-3’-methoxyphenyl)-1,2,3,4-tetrahydro-1,6-dimethyl-2-thioxopyrimidine-5-carboxylate 4j

White powder; Yield: 74%; Mp = 160°C; IR (ATR) *v*:3385, 1714, 1624, 1602 cm^{-1};^1H NMR (200 MHz, DMSO-d_6): δ 9.93 (d, *J* = 4.7 Hz, 2H), 8.07 – 8.11 (m, 2H), 7.75 (t, *J* = 7.3 Hz, 1H), 7.60 (t, *J* = 7.5 Hz, 2H), 7.21 (d, *J* = 8.2 Hz, 1H), 7.01 (d, *J* = 1.8 Hz, 1H), 6.80 (dd, *J* = 8.2, 1.9 Hz, 1H), 5.26 (d, *J* = 4.6 Hz, 1H), 3.73 (s, 3H), 3.68 (s, 3H),3.51 (s, 3H), 2.55 (s, 3H) ppm;^{13}C NMR (50 MHz, DMSO-d_6):δ 178.3, 165.8, 164.0, 150.9, 148.5, 141.2, 138.8, 134.1, 129.8, 129.1, 128.6, 123.2, 117.8, 110.7, 105.1, 66.5, 55.9, 52.0, 51.7, 36.4, 16.5 ppm; ESI-MS (m/z): 427 $[M + H]^+$.

methyl 4-(4’-(4’’-methylbenzoyloxy)-3’-methoxyphenyl)-1,2,3,4-tetrahydro-1,6-dimethyl-2-thioxopyrimidine-5-carboxylate 4k

White powder; Yield: 34%; Mp = 186°C; IR (ATR) *v*:3338, 1722, 1679, 1504 cm^{-1};^1H NMR (200 MHz, DMSO-d_6): δ 9.94 (d, *J* = 4.7 Hz, 1H), 7.99 (d, *J* = 8.2 Hz, 2H), 7.40 (d, *J* = 8.1 Hz, 2H), 7.19 (d, *J* = 8.1 Hz, 1H), 7.01 (d, *J* = 1.9 Hz, 1H), 6.80 (dd, *J* = 8.2, 1.9 Hz, 1H), 5.26 (d, *J* = 4.6 Hz, 1H), 3.73 (s, 3H), 3.68 (s, 3H), 3.51 (s, 3H), 2.55 (s, 3H), 2.42 (s, 3H) ppm;^{13}C NMR (50 MHz, DMSO-d_6):δ 178.3, 165.7, 164.0, 150.9, 148.4, 144.5, 141.1, 138.9, 129.9, 129.6, 125.9, 123.2, 117.8, 110.7, 105.2, 55.8, 52.0, 51.7, 36.4, 21.4, 16.5 ppm; ESI-MS (m/z): 441 $[M + H]^+$ and 463 $[M + Na]^+$.

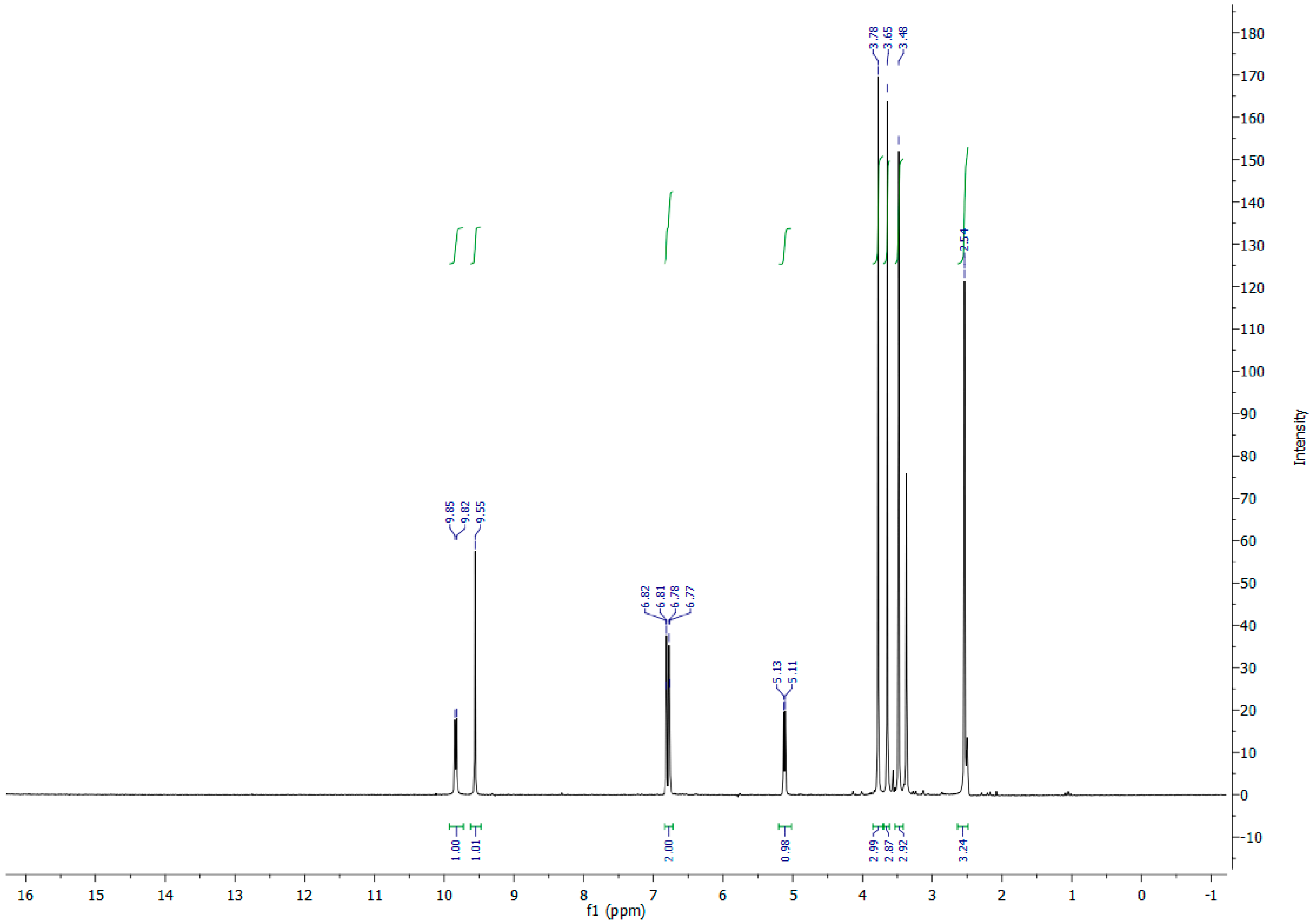

Figure S1.^{1}H NMR spectrum of **4a**

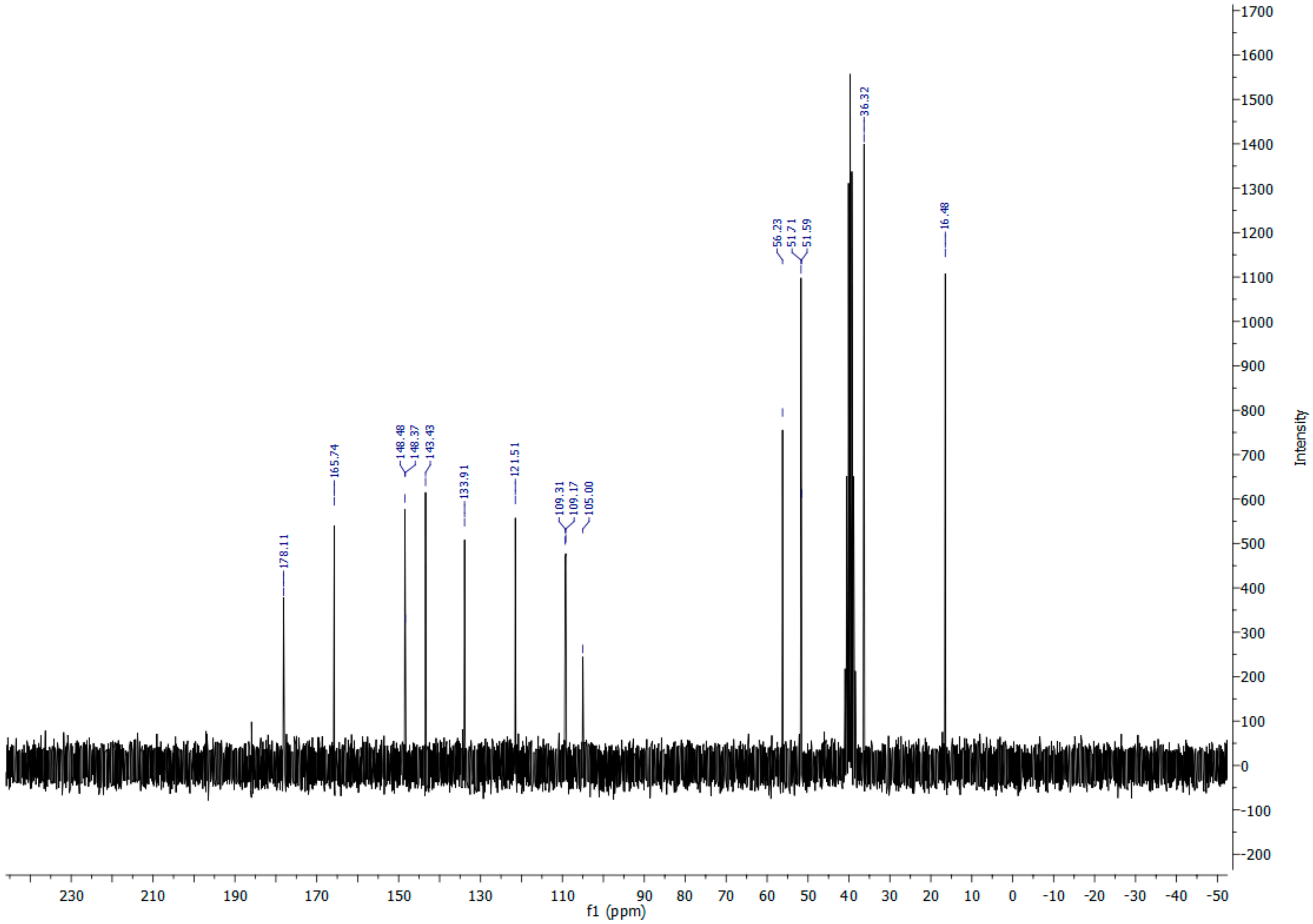

Figure S2.^{13}C NMR spectrum of **4a**

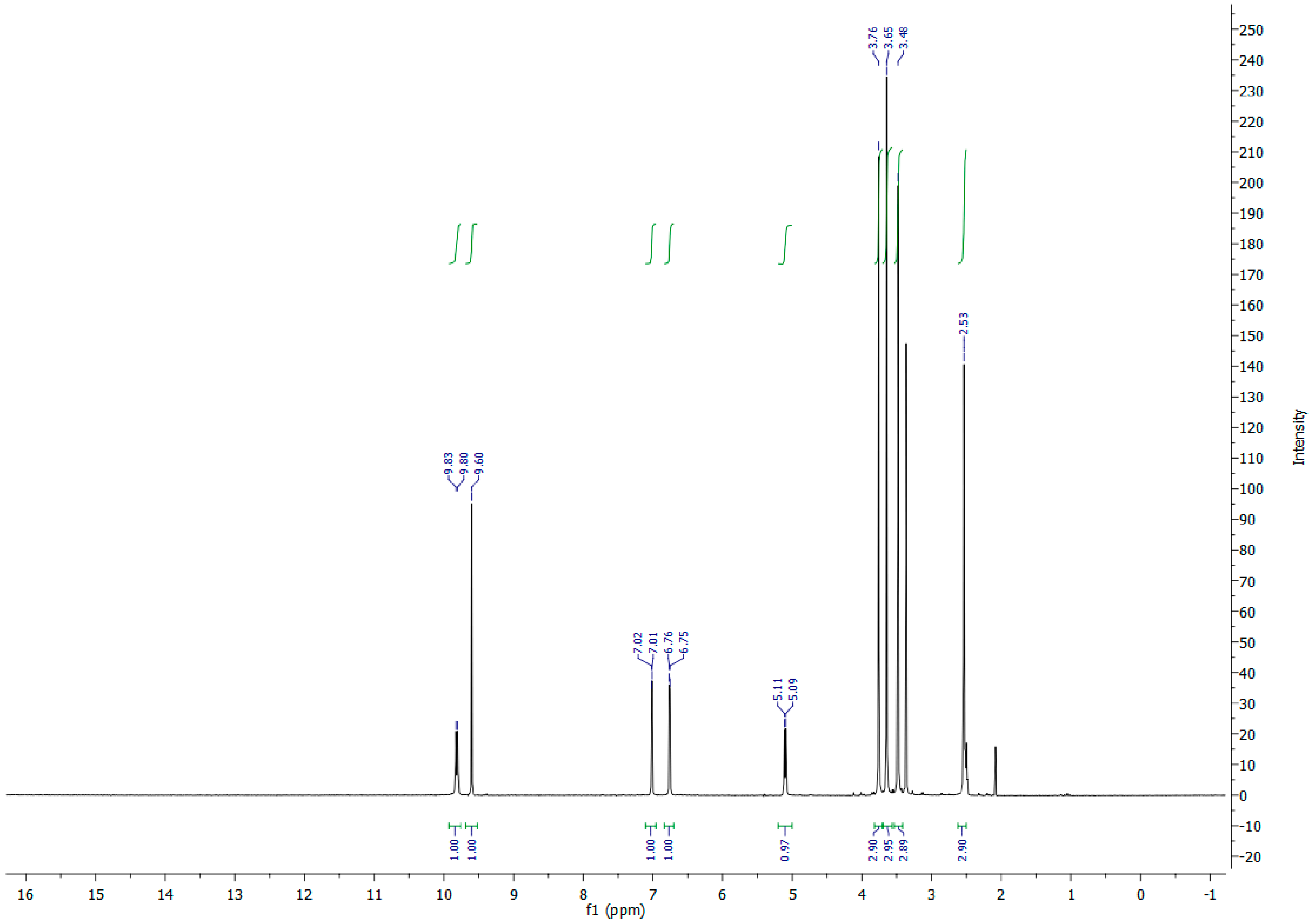

Figure S3. ^{1}H NMR spectrum of **4b**(protons at 2.1 ppm came from residual solvent-acetone)

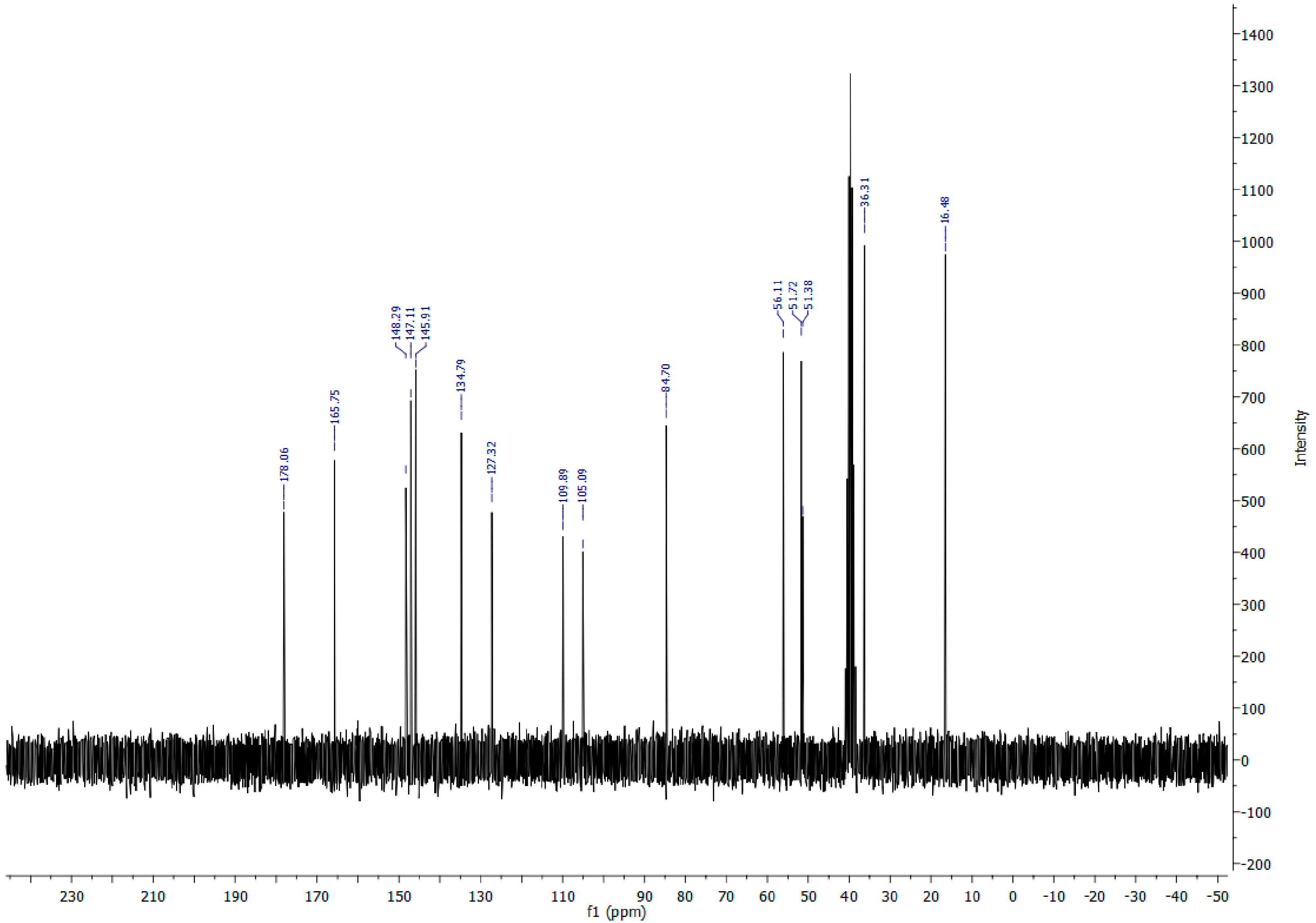

Figure S4.^{13}C NMR spectrum of **4b**

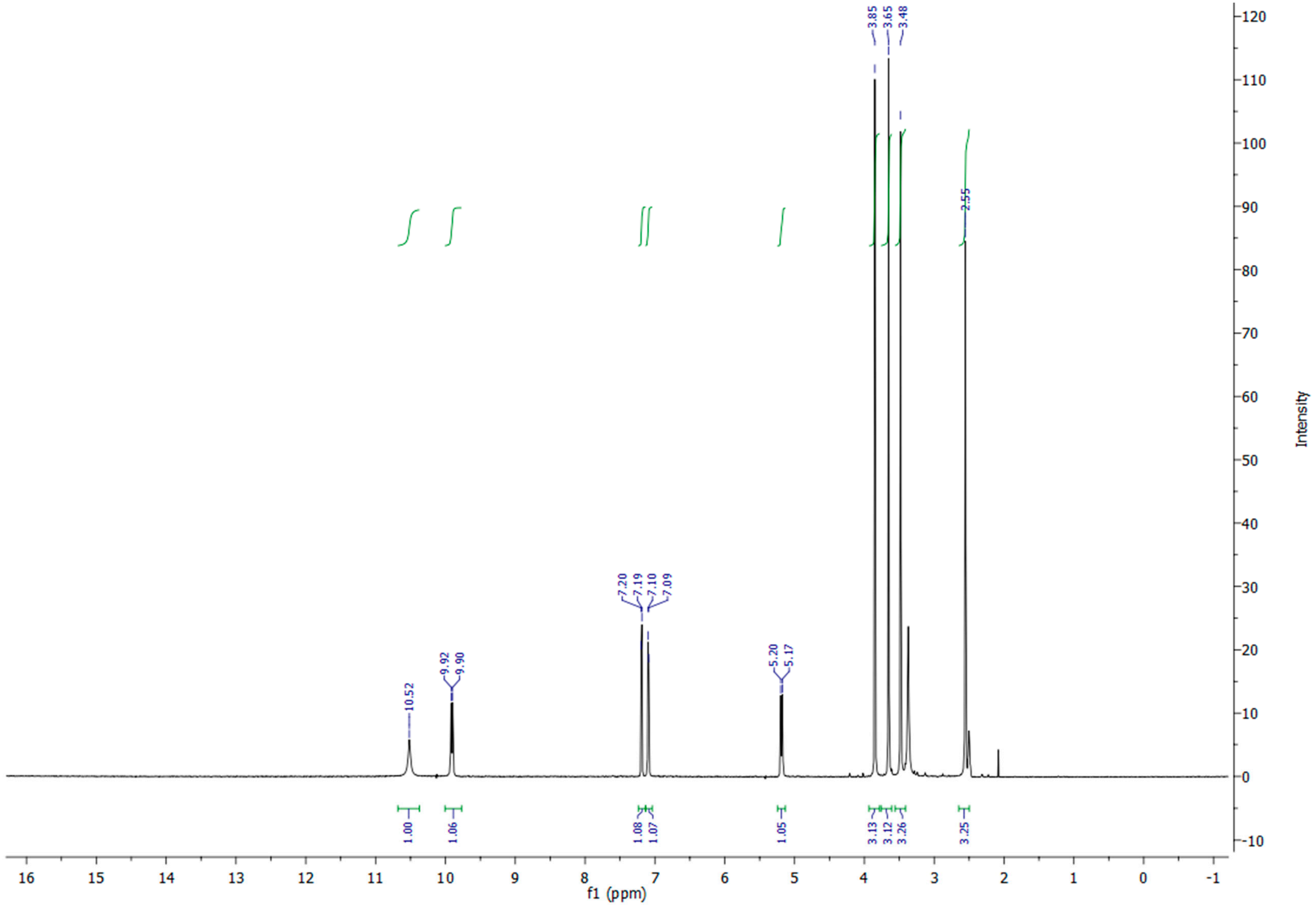

Figure S5.^{1}H NMR spectrum of **4c** (protons at 2.1 ppm came from residual solvent-acetone)

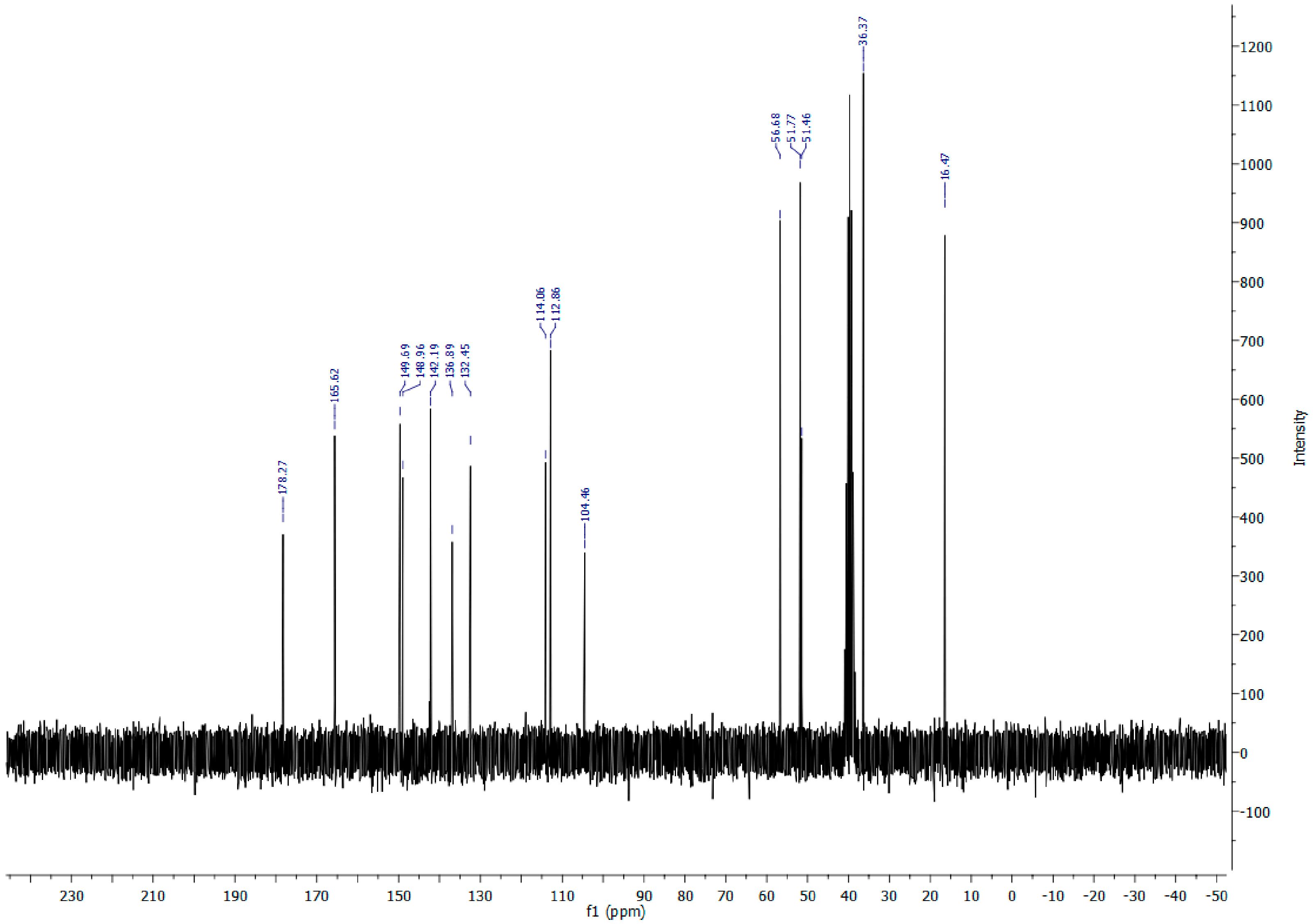

Figure S6.^{13}C NMR spectrum of **4c**

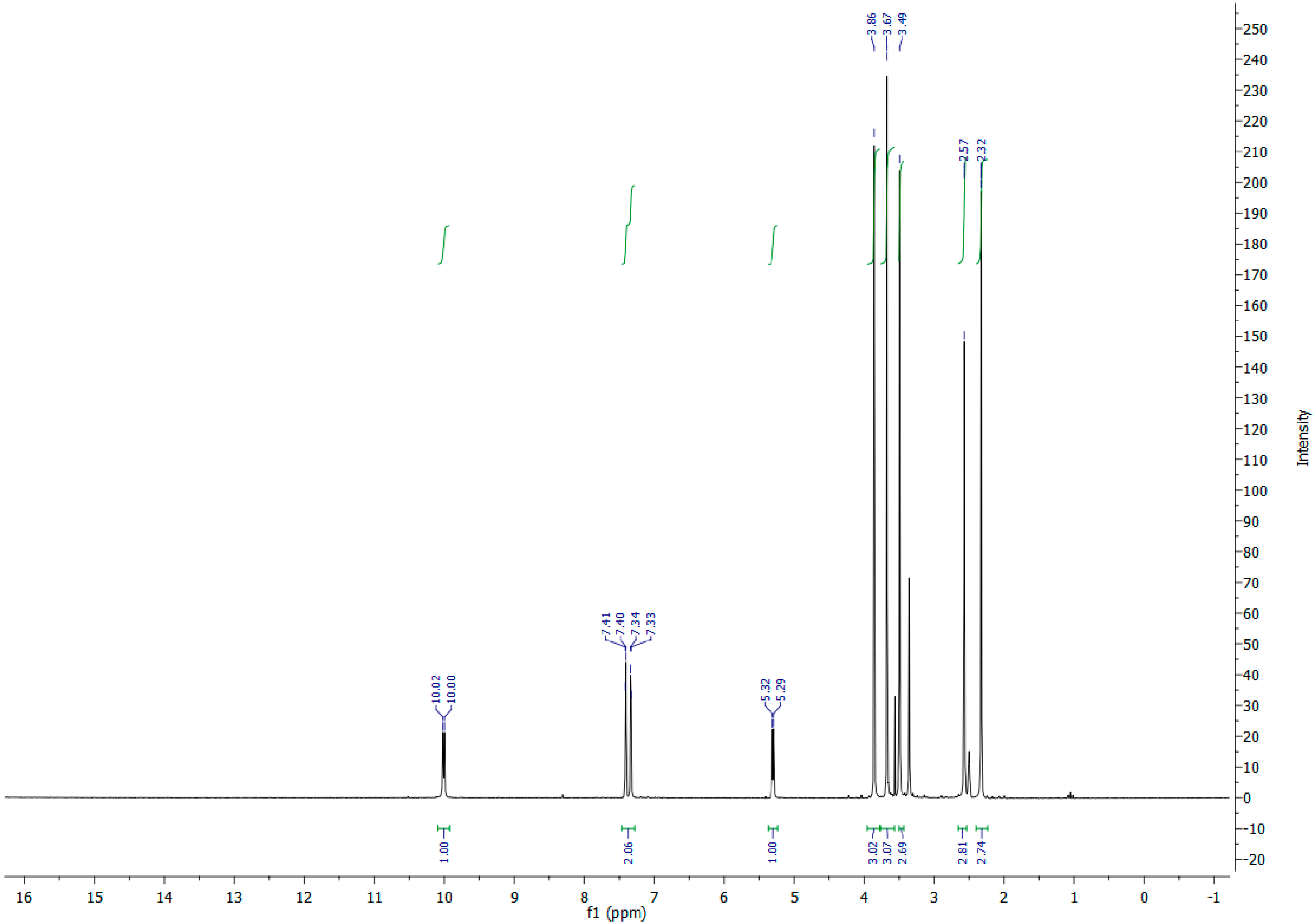

Figure S7.^{1}H NMR spectrum of **4d**(protons at 3.56 ppm came from residual solvent-dioxane)

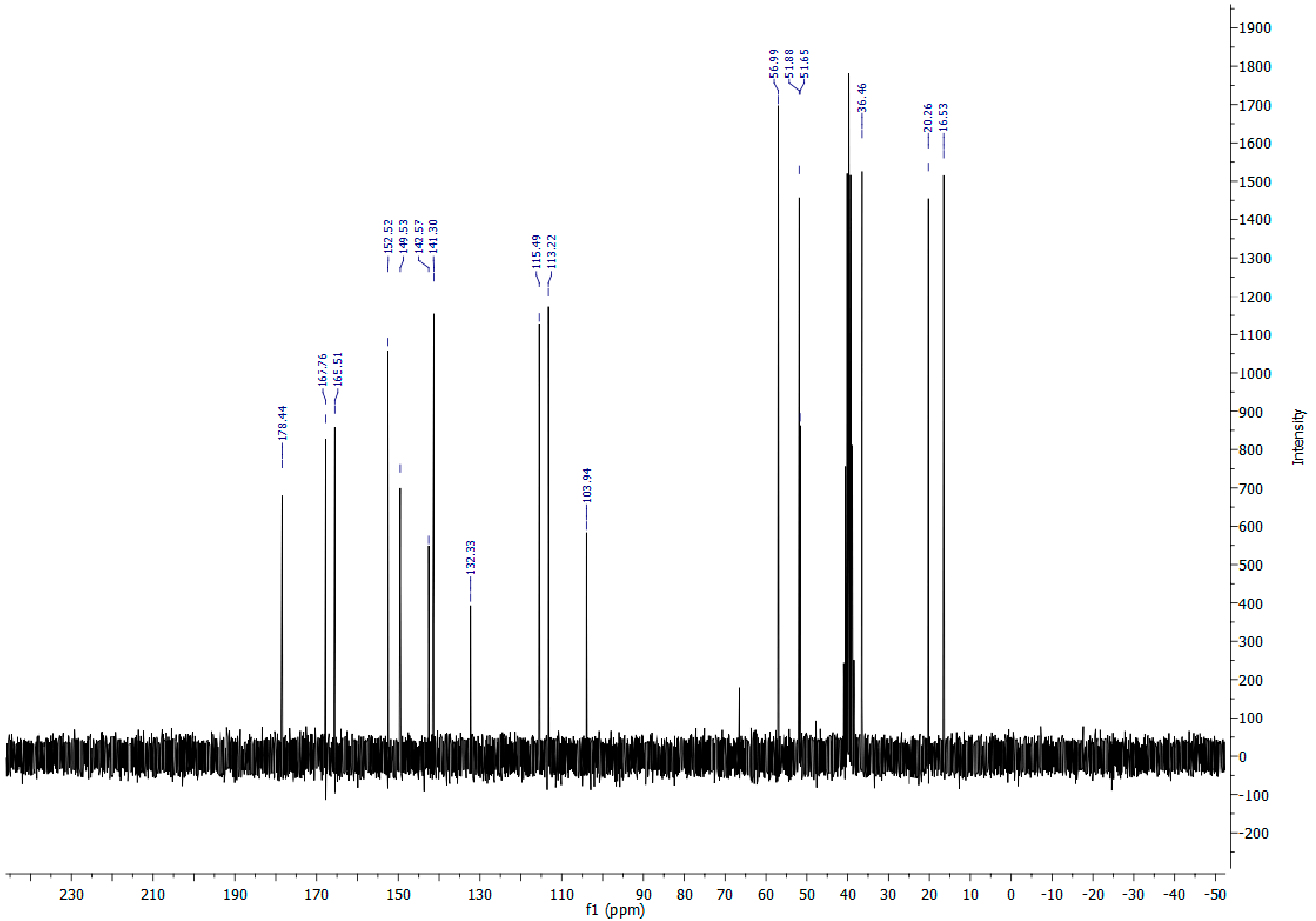

Figure S8.^{13}C NMR spectrum of **4d**(carbon at 66.5 ppm came from residual solvent-dioxane)

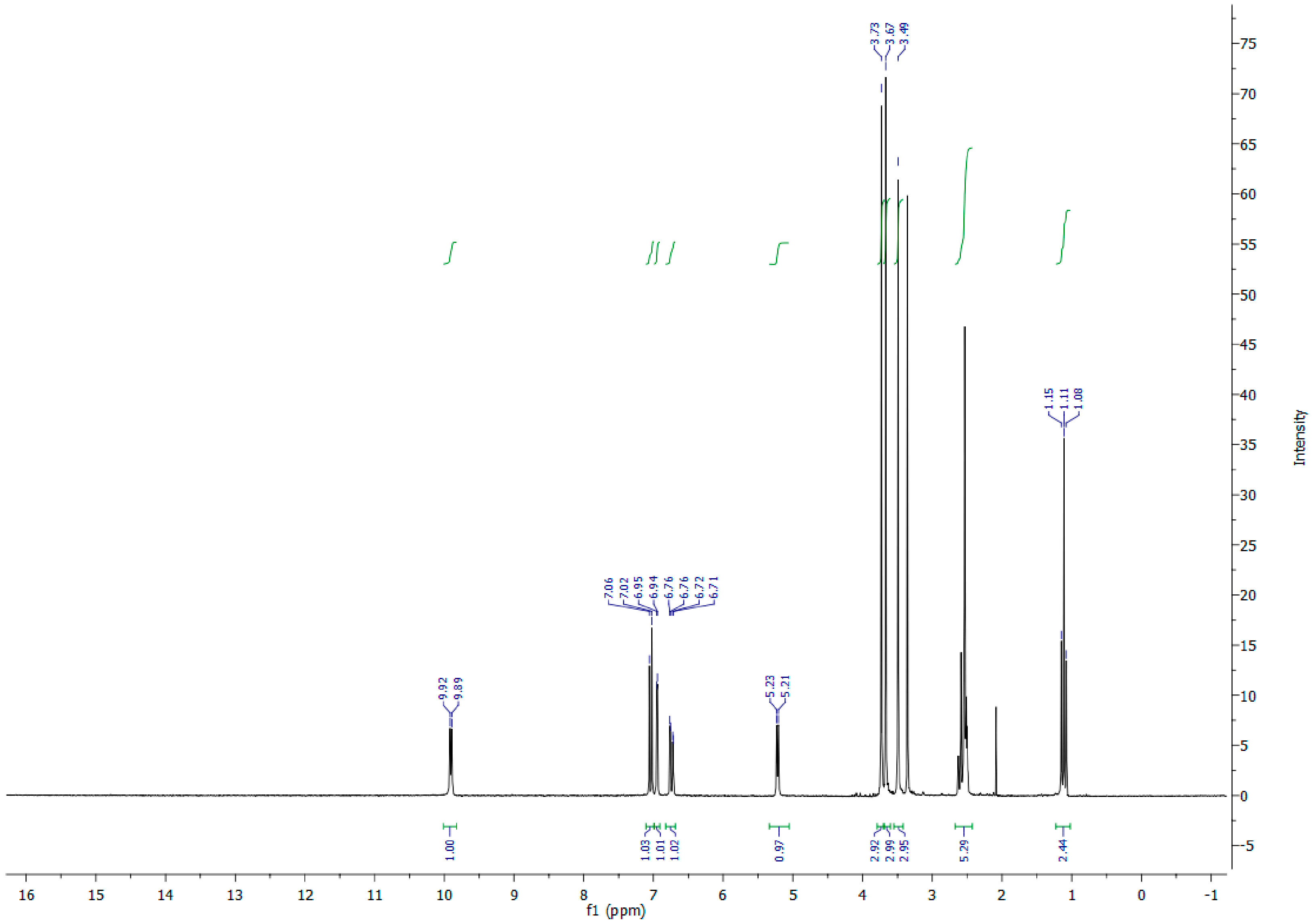

Figure S9.^{1}H NMR spectrum of **4e**(protons at 2.1 ppm came from residual solvent-acetone)

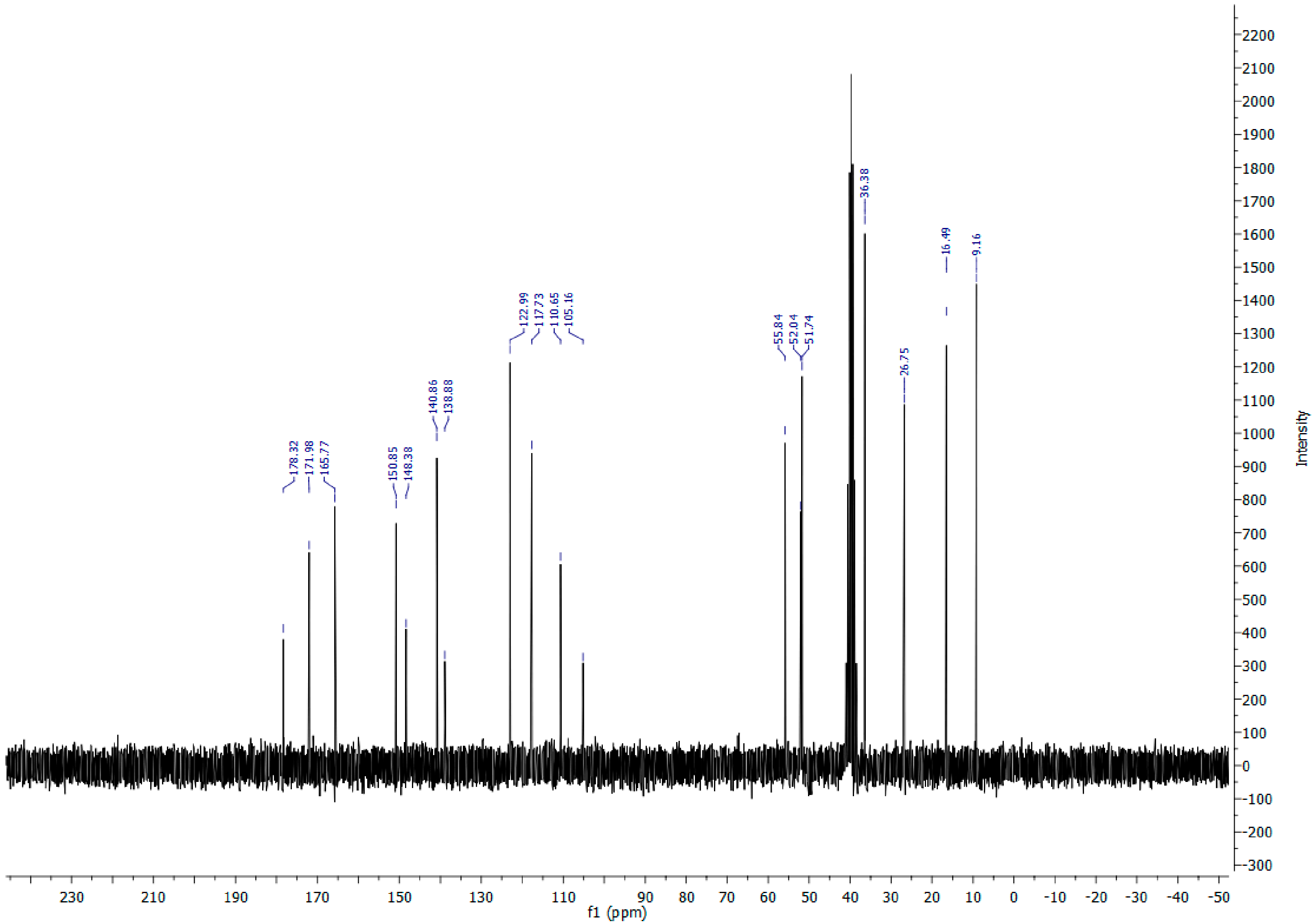

Figure S10. ^{13}C NMR spectrum of **4e**

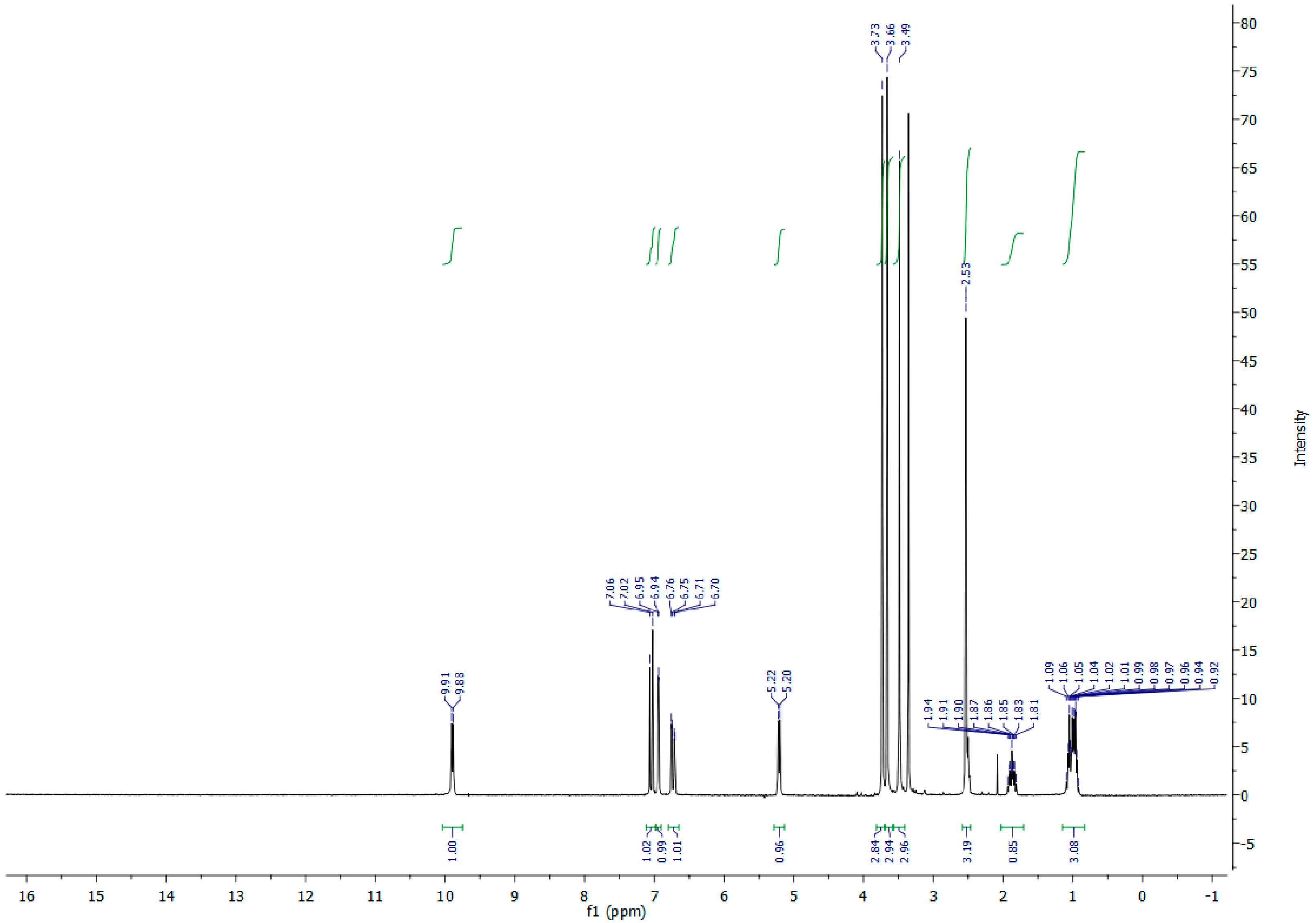

Figure S11. ^{1}H NMR spectrum of **4f**(protons at 2.1 ppm came from residual solvent-acetone)

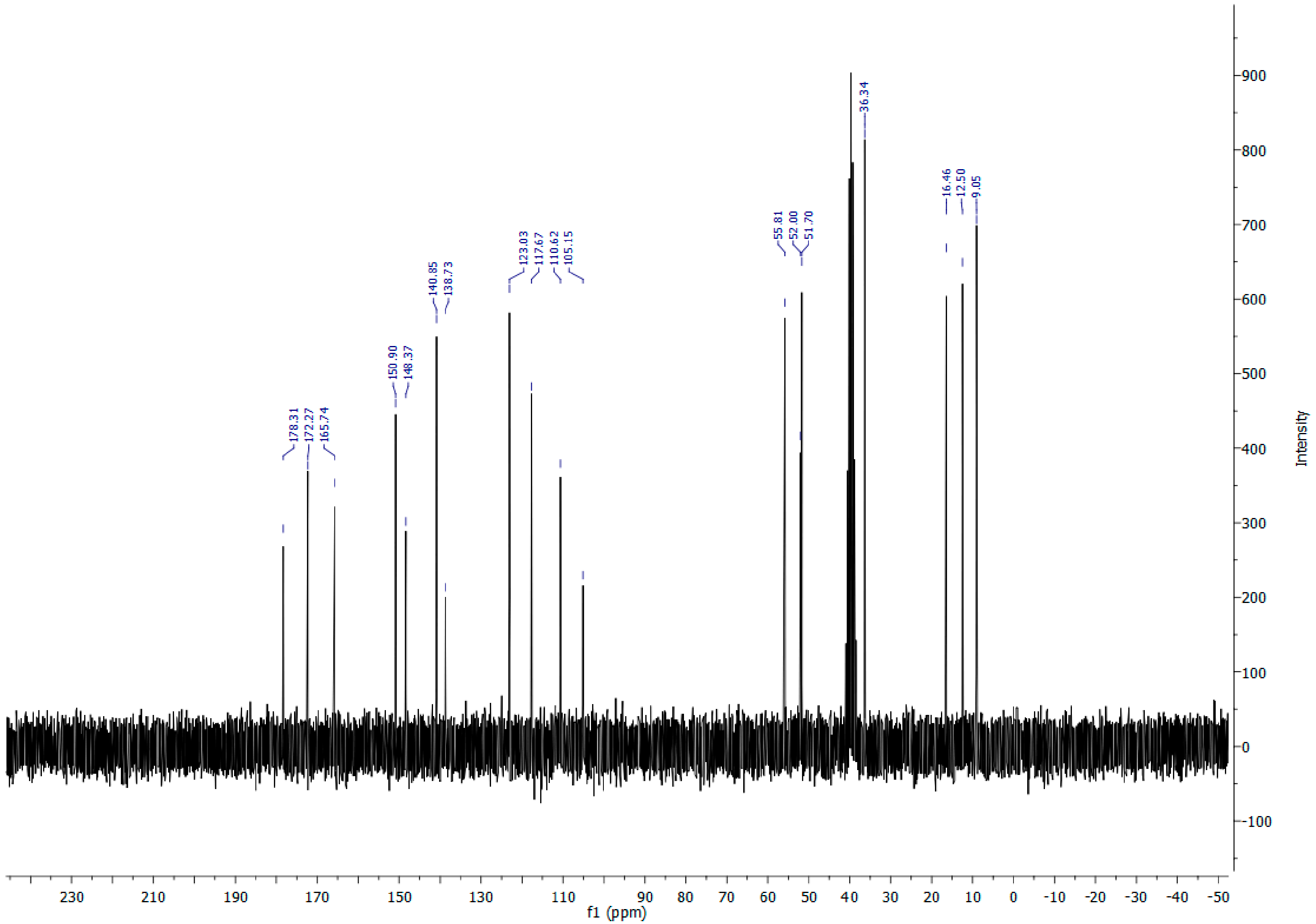

Figure S12. ^{13}C NMR spectrum of **4f**

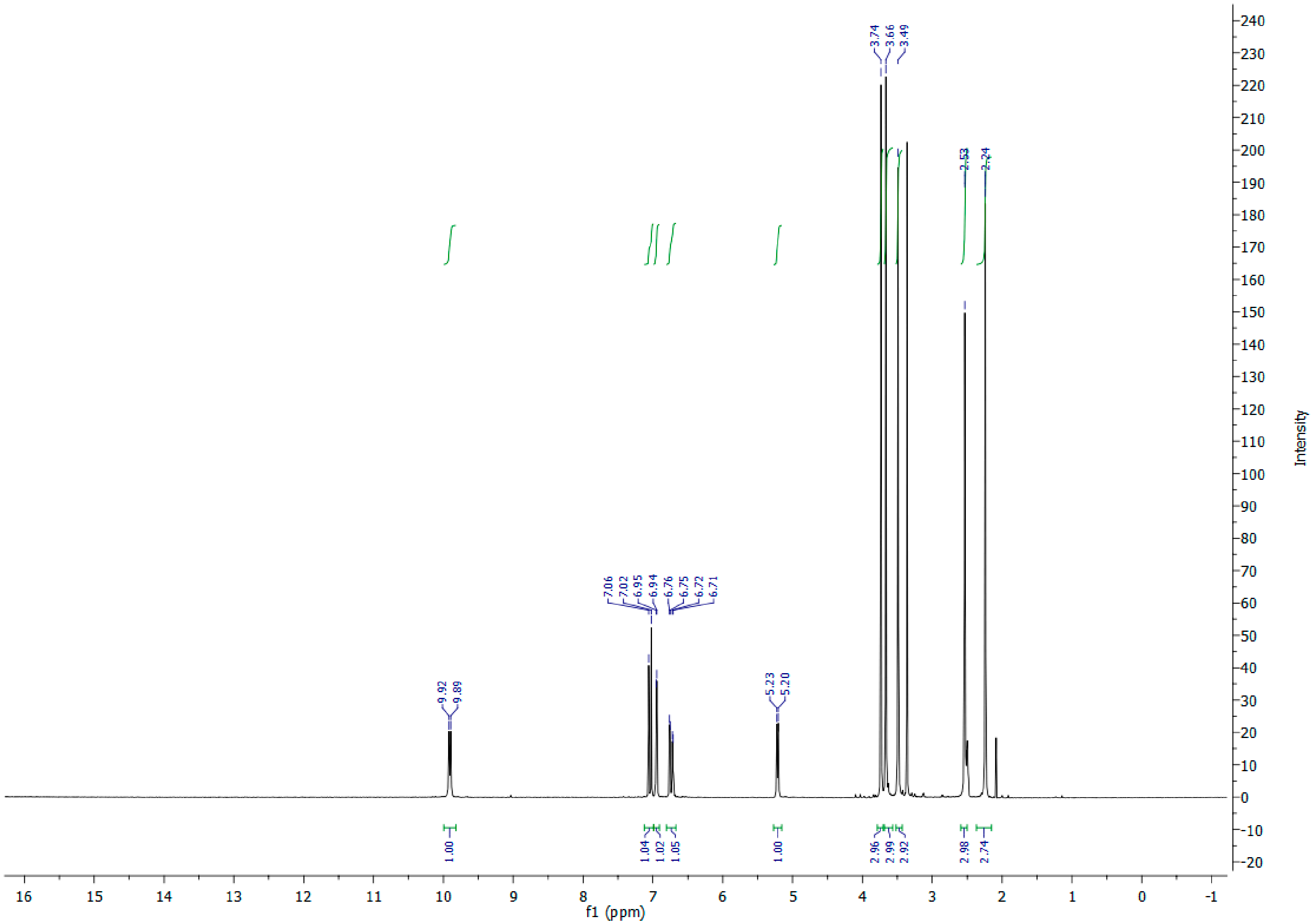

Figure S13. ¹H NMR spectrum of **4g** (protons at 2.1 ppm came from residual solvent-acetone)

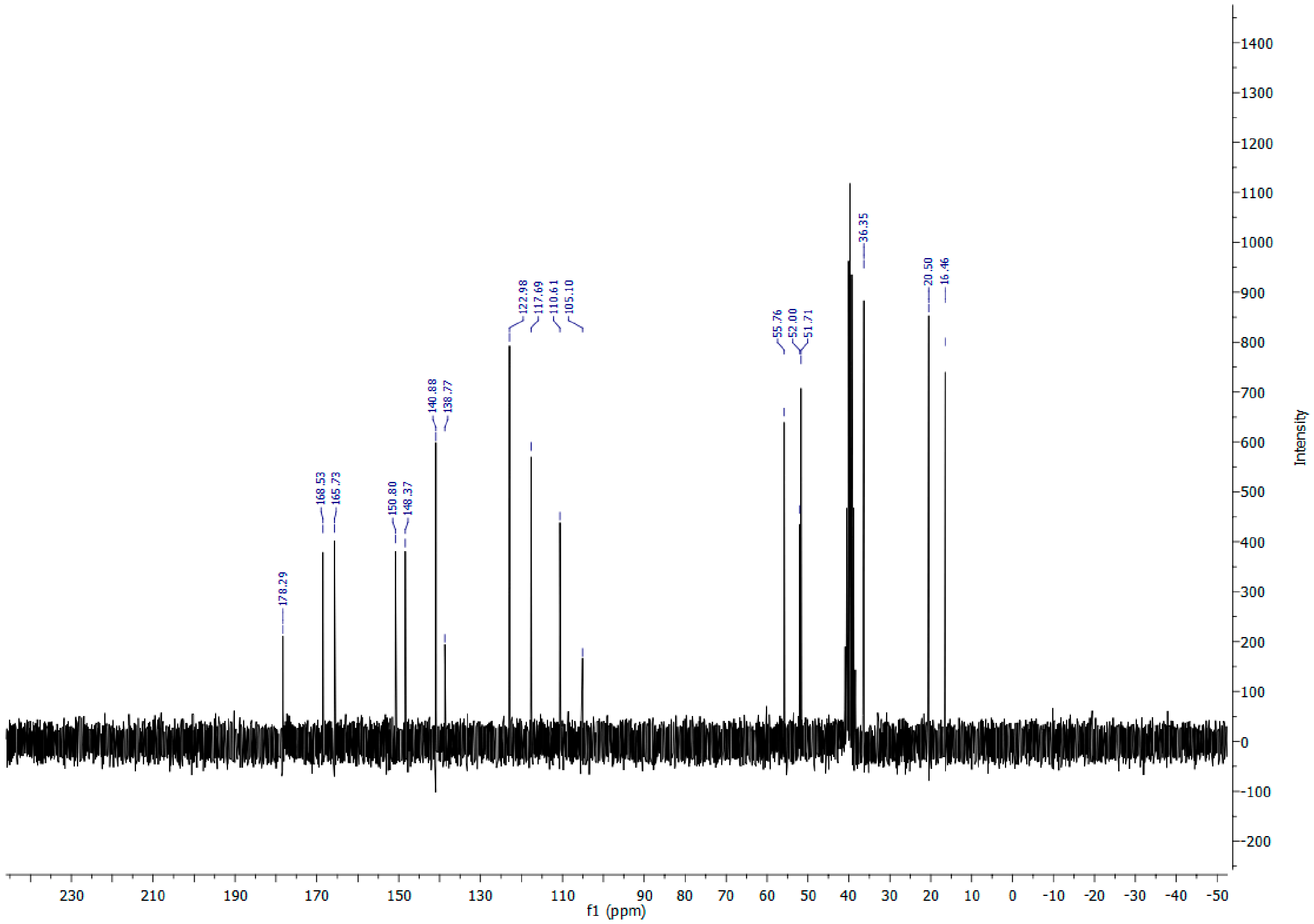

Figure S14.^{13}C NMR spectrum of **4g**

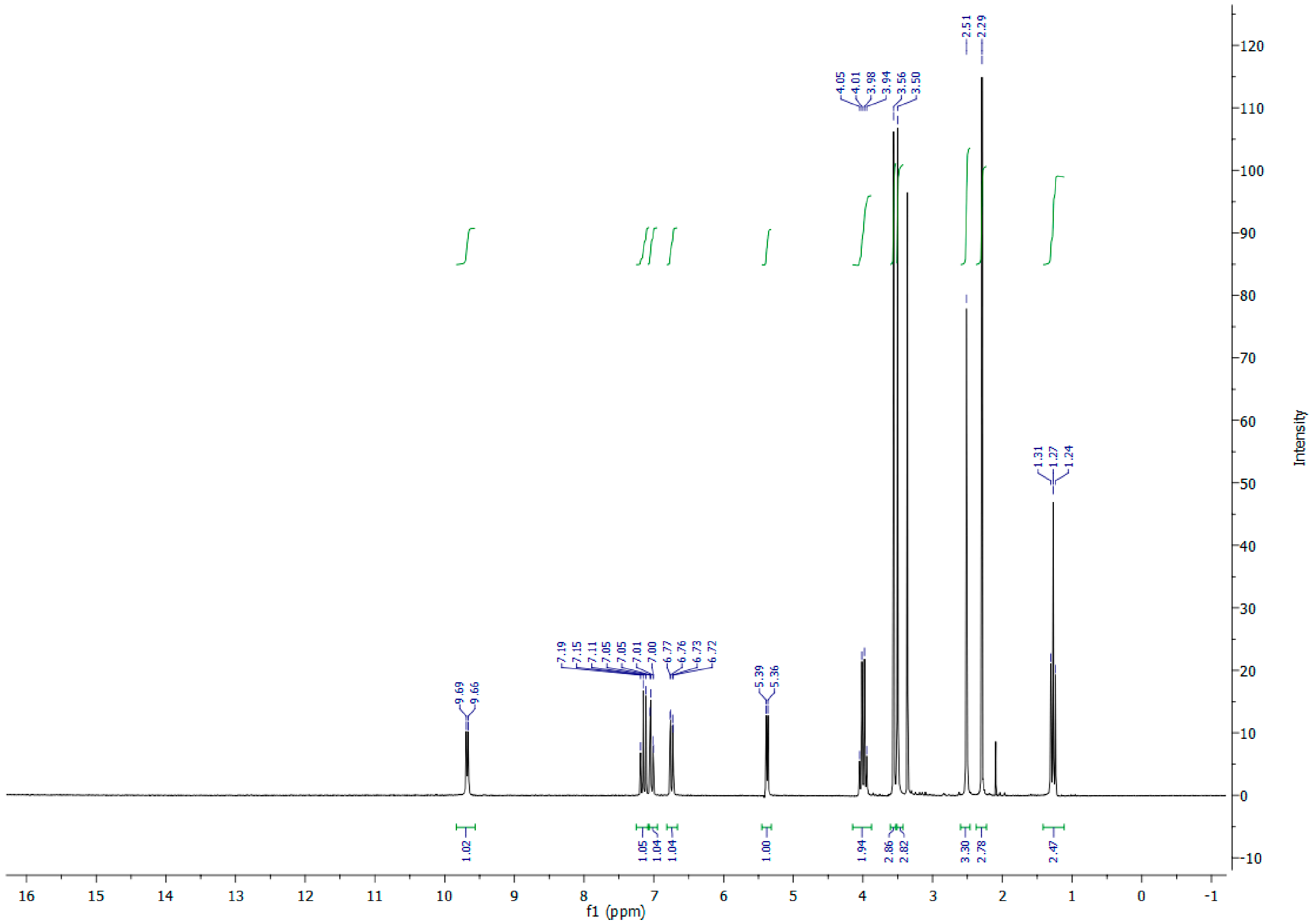

Figure S15.^{1}H NMR spectrum of **4h**(protons at 2.1 ppm came from residual solvent-acetone)

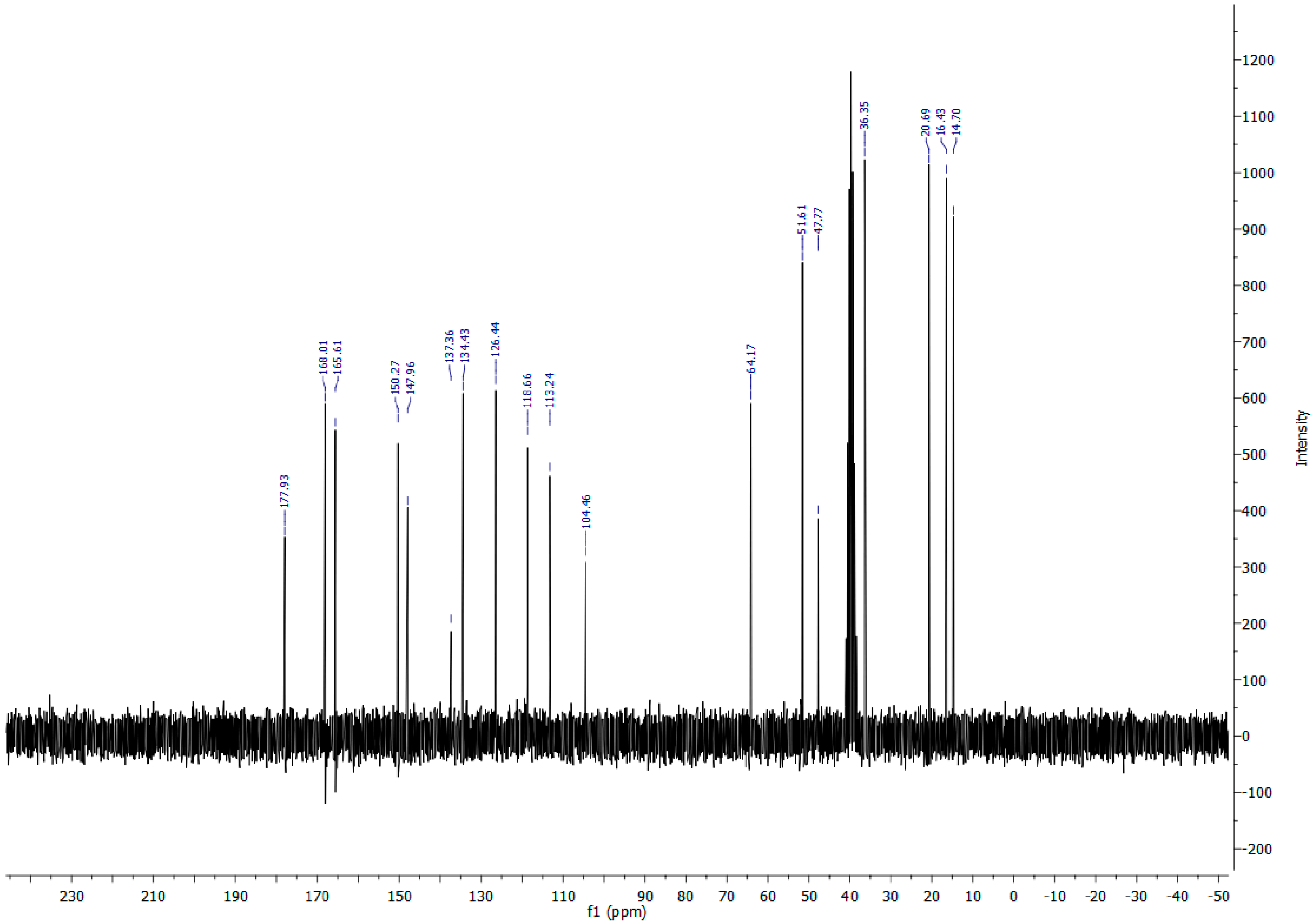

Figure S16.^{13}C NMR spectrum of **4h**

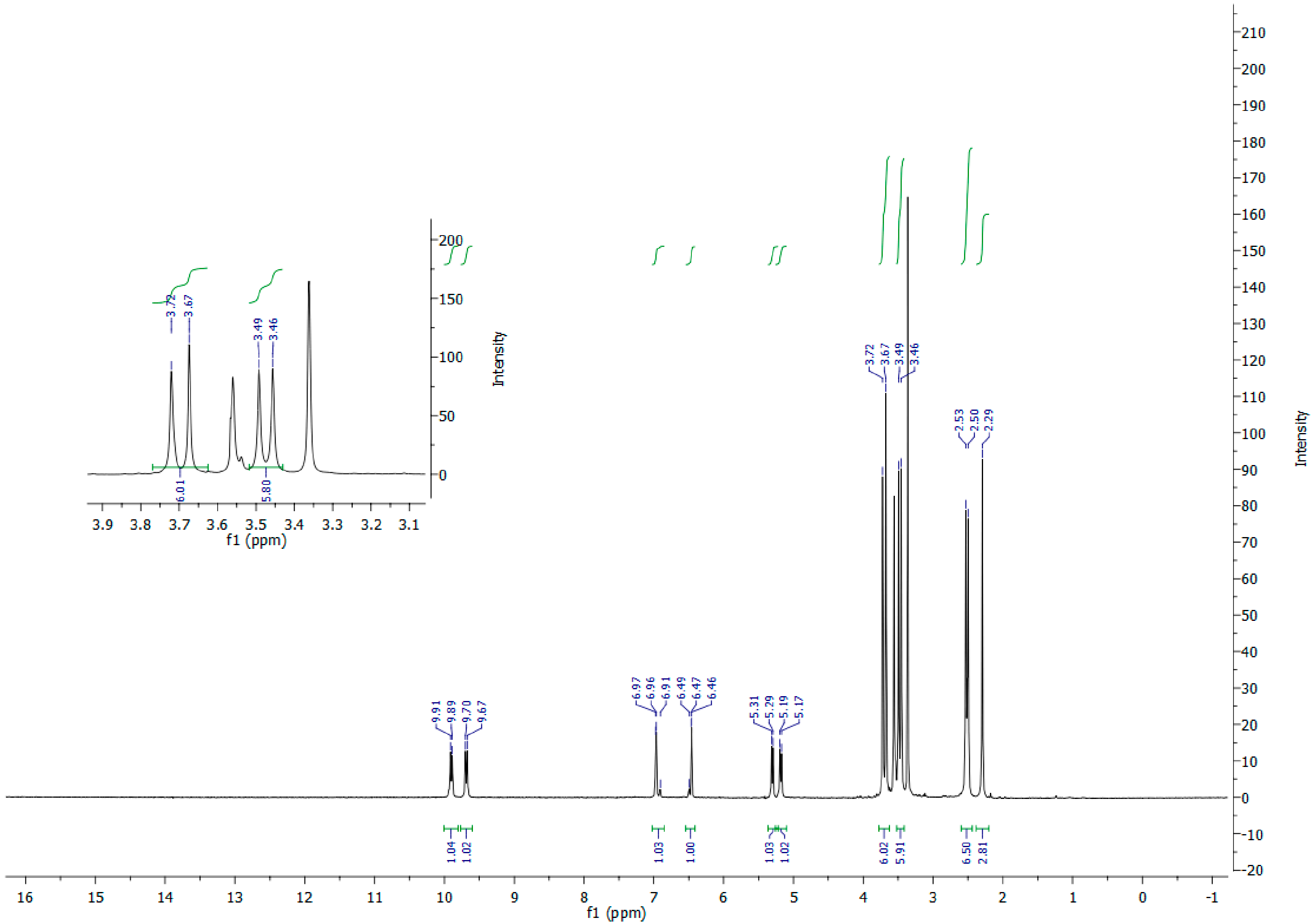

Figure S17.^{1}H NMR spectrum of **4i** (protons at 3.57 ppm is from dioxane)

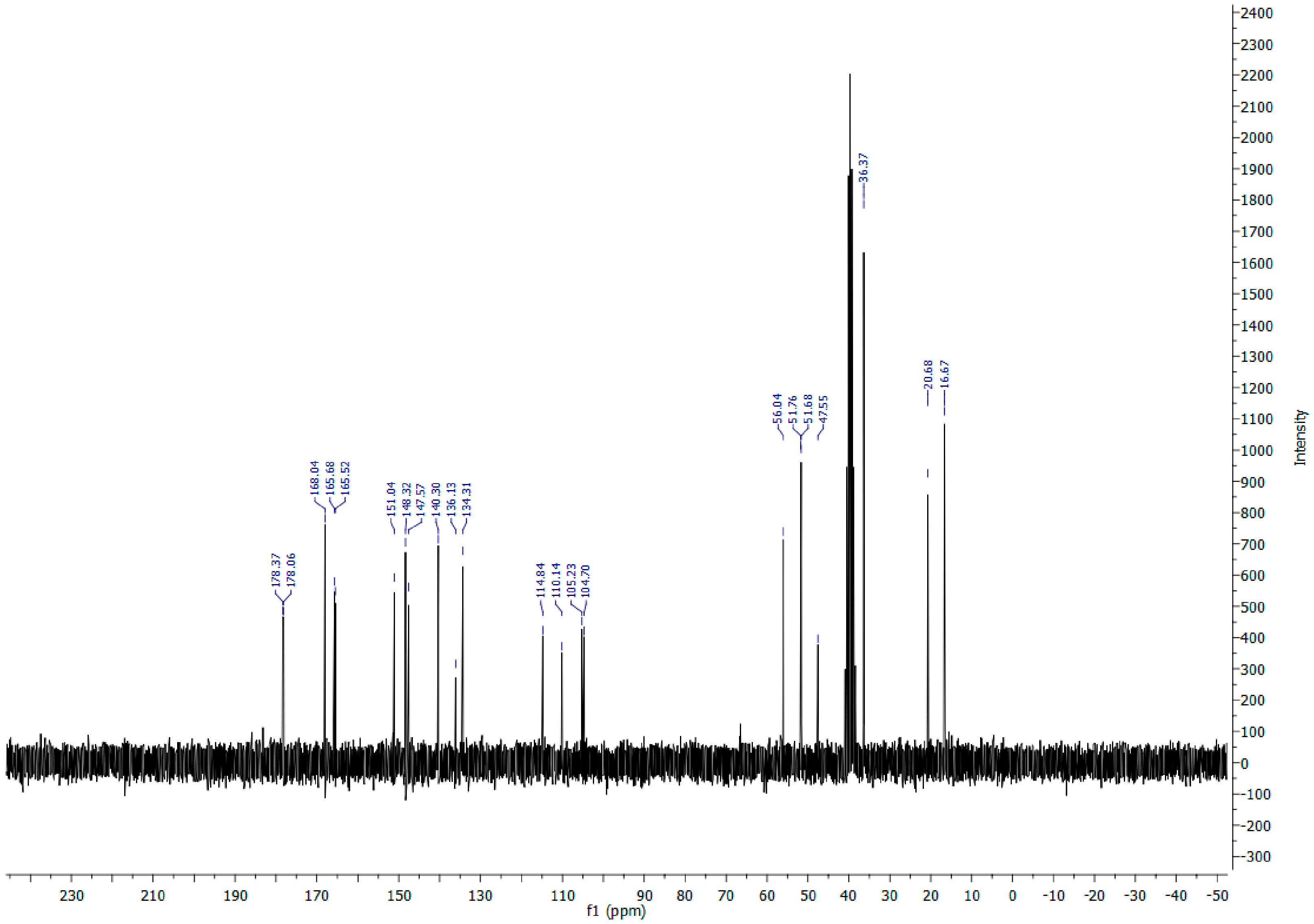

Figure S18.^{13}C NMR spectrum of **4i** (carbon at 66.4 ppm is from dioxane)

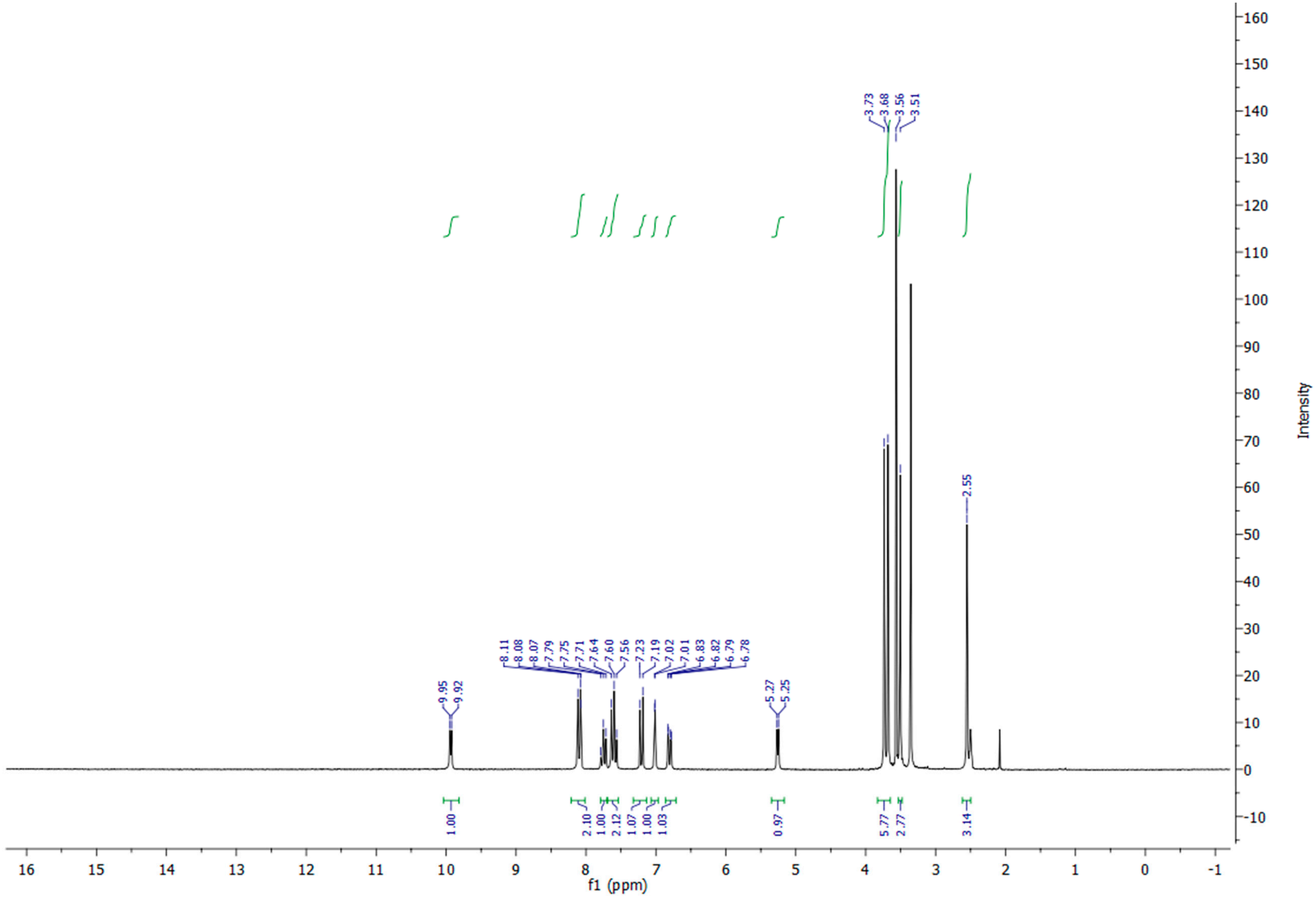

Figure S19. ^{1}H NMR spectrum of **4j** (protons at 3.56 ppm is from dioxane)

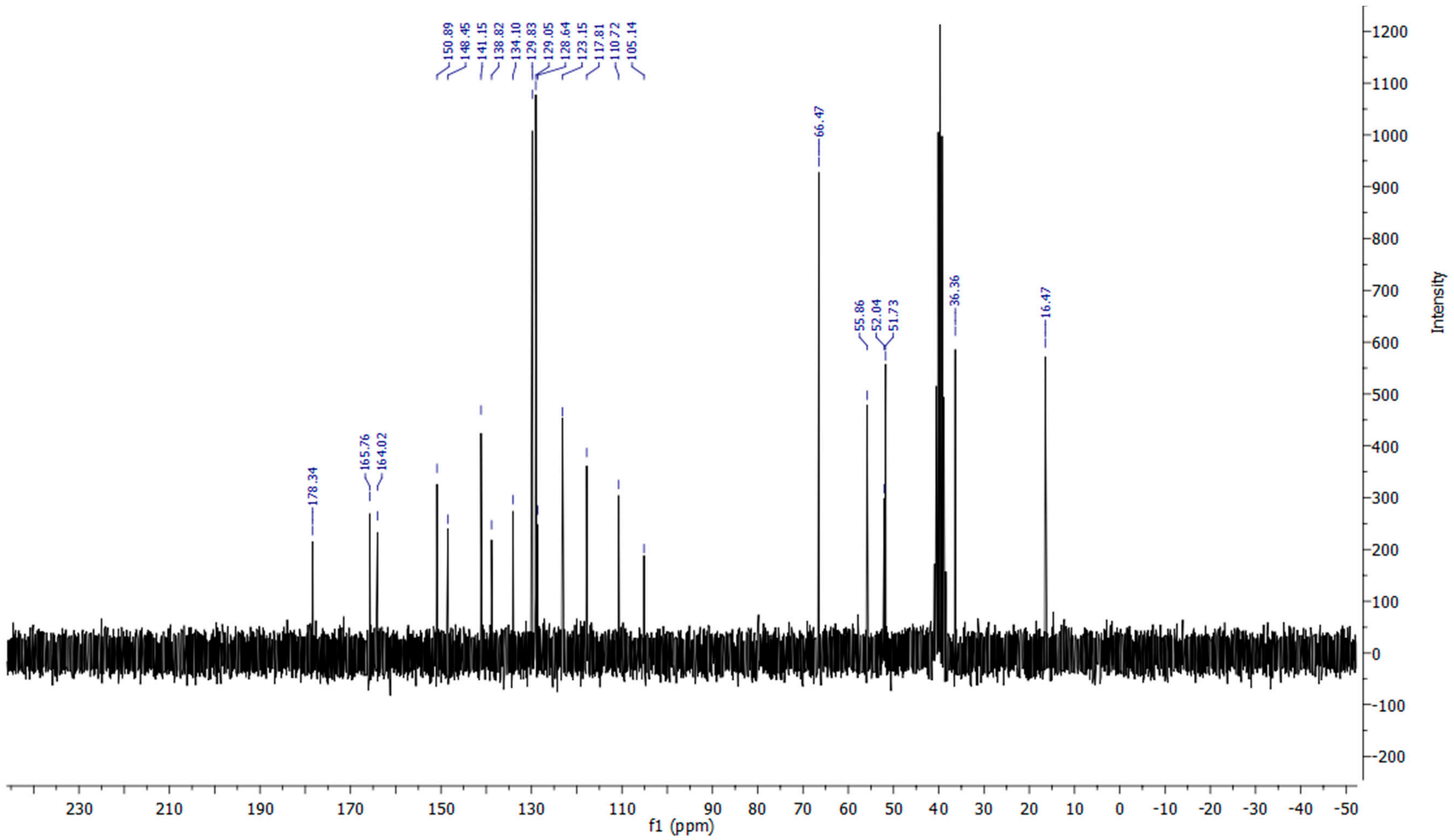

Figure S20. ^{13}C NMR spectrum of **4j** (carbon at 66.4 ppm is from dioxane)

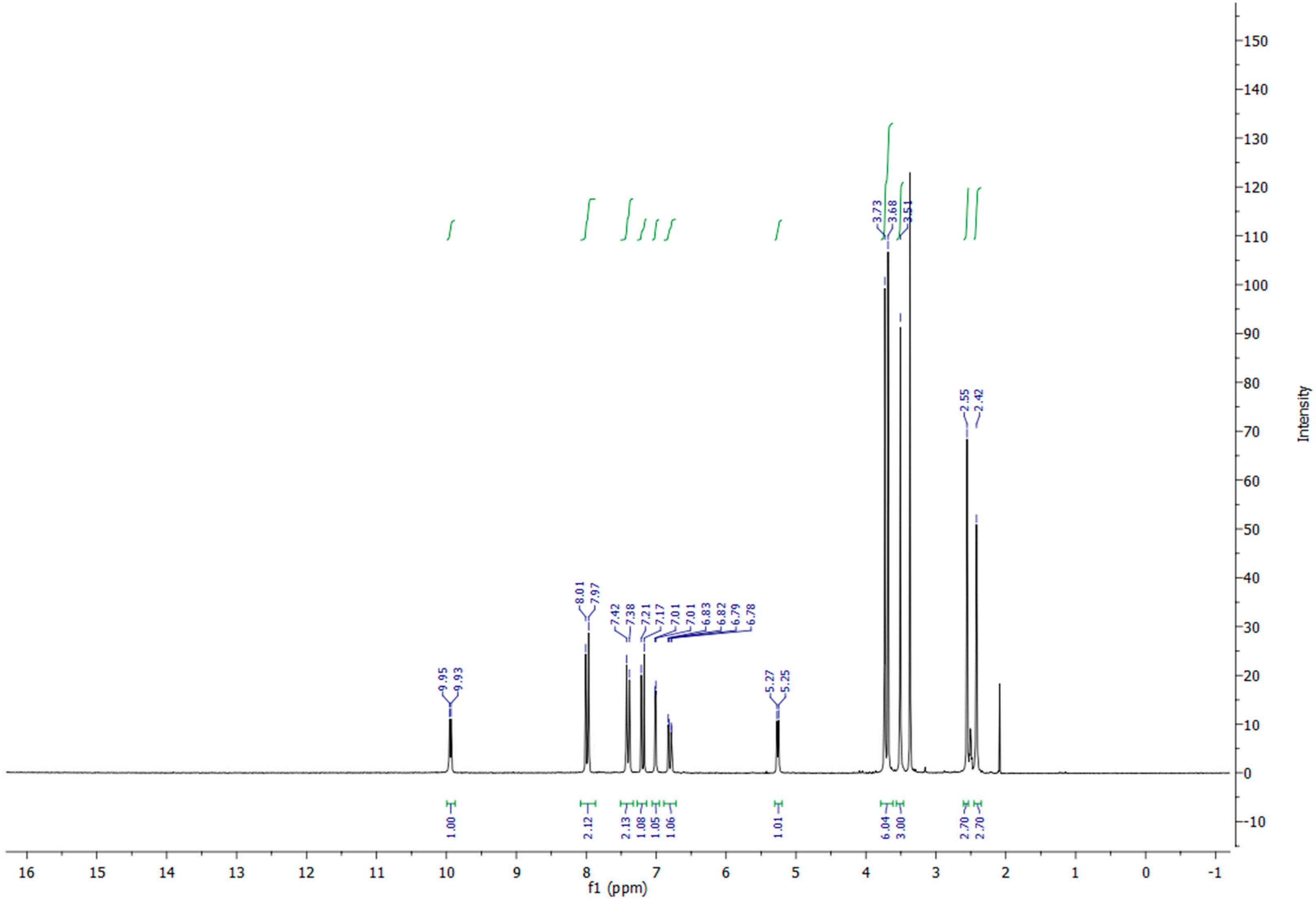

Figure S21.^{1}H NMR spectrum of **4k**

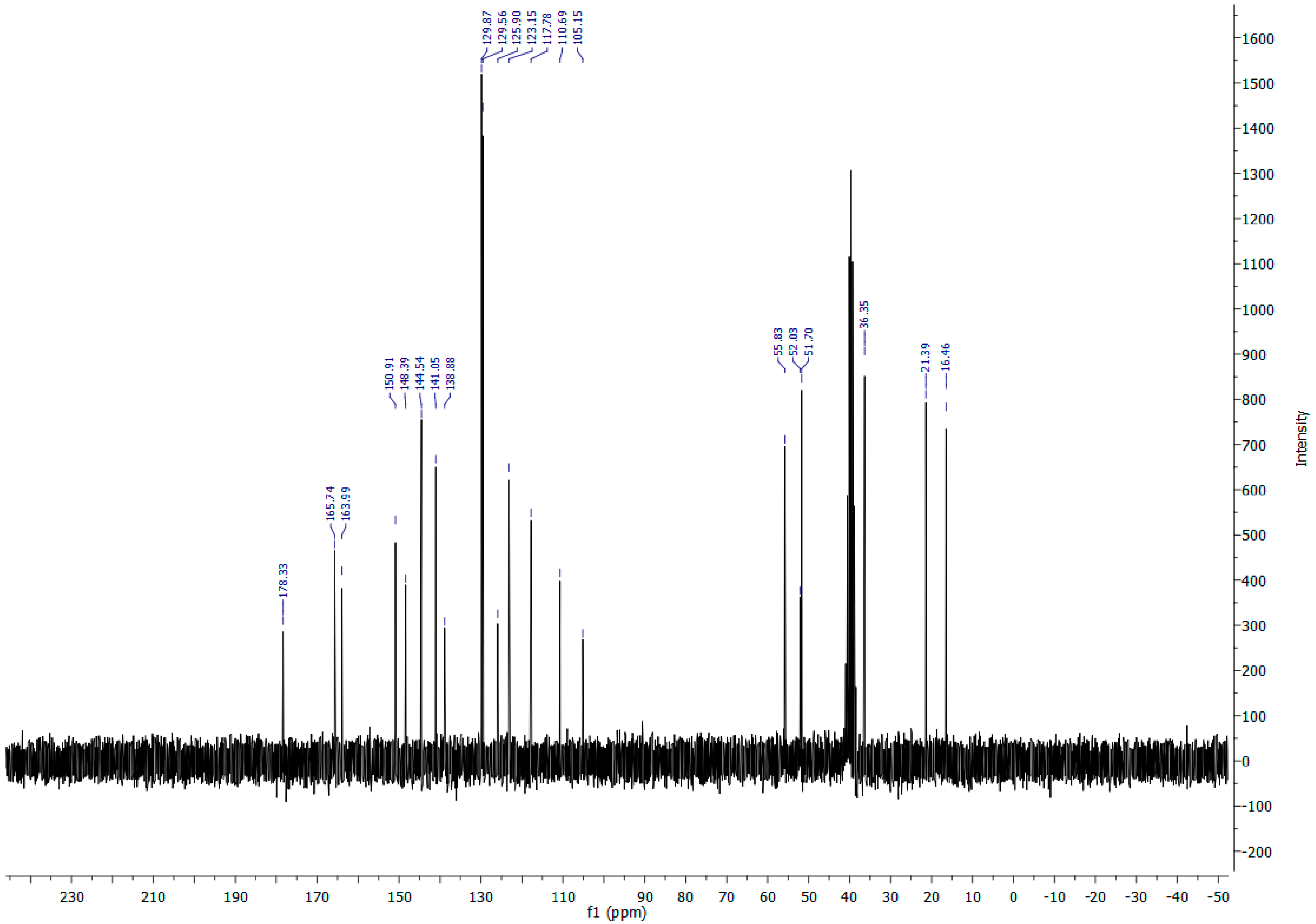

Figure S22.^{13}C NMR spectrum of **4k**

1. Sarker, S.D.; Nahar, L.; Kumarasamy, Y. Microtitre plate-based antibacterial assay incorporating resazurin as an indicator of cell growth, and its application in the in vitro antibacterial screening of phytochemicals. *Methods.* **2007**, *42*, 321-324. https://doi.org/10.1016/j.ymeth.2007.01.006
2. Mosmann, T.Rapid colorimetric assay for cellular growth and survival: application to proliferation and cytotoxicity assays. *J. Immunol. Methods.***1983**, *65*, 55-63. https://doi.org/10.1016/0022-1759(83)90303-4
3. Ohno, M.; Abe, T. Rapid colorimetric assay for the quantification of leukemia inhibitory factor (LIF) and interleukin-6 (IL-6). *J. Immunol. Methods.***1991**, *145*, 199-203. https://doi.org/10.1016/0022-1759(91)90327-c
4. Ormerod, M.G. Flow cytometry. A practical approach. Oxford University Press 2000.
5. McCue, P.; Kwon, Y.I.; Shetty, C. Anti-amylase, anti-glucosidase and anti-angiotensin i-converting enzyme potential of selected foods. *J. Food Biochem.* **2005**, *29*, 278-294. https://doi.org/10.1111/j.1745-4514.2005.00020.x
6. Grozdanić, N.; Zdunić, G.; Šavikin, K.; Đuričić, I.; Kosanić, M.; Mačić, V.; Matić, I.Z.; Stanojković, T. Seasonal variation in biopharmaceutical activity and fatty acid content of endemic Fucus virsoidesalgae from Adriatic sea. *Acta Poloniae Pharmaceutica.* **2019**, *76*, 833-844. https://doi.org/ 10.32383/appdr/108920
7. Rigaku, O.D.CrysAlis PRO. Rigaku Oxford Diffraction Ltd, Yarnton, Oxfordshire, England. 2015
8. Blessing, R.H. An empirical correction for absorption anisotropy. *Acta Crystallogr. A.* **1995**, *51*, 33–38. https://doi.org/10.1107/s0108767394005726
9. Sheldrick, G.M. SHELXT – Integrated Space-Group and Crystal-Structure Determination. *Acta Crystallogr. A: Found. Adv.* **2015,** *71*, 3–8. https://doi.org/10.1107/S2053273314026370
10. Sheldrick, G.M. Crystal Structure Refinement with SHELXL, *Acta Crystallogr. Sect. C Struct. Chem.* **2015**, *71*, 3–8. http://dx.doi.org/10.1107/S2053229614024218
11. Bruno, I.J.; Cole, J.C.; Edgington, P.R.; Kessler, M.K.; Macrae, C.F.; McCabe, P.; Pearson, J.; Taylor, R. New software for searching the Cambridge Structural Database and visualizing crystal structures. *Acta. Crystallogr. B. Struct. Sci. Cryst. Eng. Mater.* **2002**, *58*, 389-397. https://doi.org/10.1107/s0108768102003324
12. Dolomanov, O. V. ; Bourhis, L.J. ; Gildea, R.J.; Howard, J.A.K.; Puschmann, H. OLEX2: A complete structure solution, refinement and analysis program. J. *Appl. Cryst.* **2009**, *42*, 339–341. https://doi.org/10.1107/S0021889808042726
