# Supplementary material for: Synthesis, Characterization, and Biological Evaluation of Tetrahydropyrimidines: Dual-Activity and Mechanism of Action
Source: Pharmaceutics. 2022 Oct 21;14(10):2254. doi: 10.3390/pharmaceutics14102254 (PMC9610702; doi:10.3390/pharmaceutics14102254)
Supplement: Supplementary file 1 [file pharmaceutics-14-02254-s001.zip › Supplementary Materials_File 2_ESI-MS.pdf]

4A

SHIMADZU LabSolutions

Analysis Report

<Sample Information>

Sample Name	: 220516 NJ 1909		
Sample ID	: 220516 NJ 1909		
Data Filename	: 220516 NJ 1909.lcd		
Method Filename	: NNDKG.lcm		
Batch Filename	:		
Vial #	: 1-1	Sample Type	: Unknown
Injection Volume	: 1 uL		
Date Acquired	: 16/05/2022 12:09:58	Acquired by	: System Administrator
Date Processed	: 16/05/2022 12:11:12	Processed by	: System Administrator

MS Spectrum

R.Time:0.433(Scan#:)
MassPeaks:60
Segment 1 - Event 1

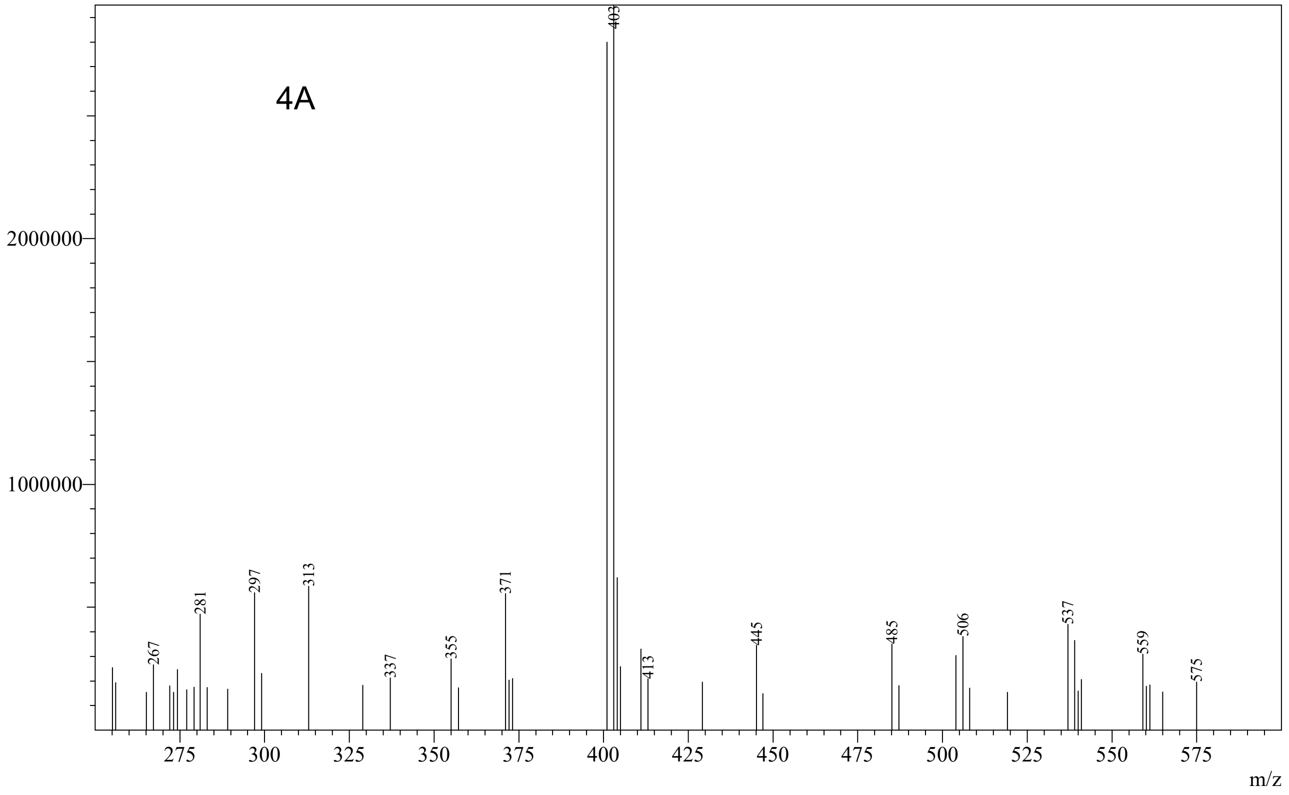

MS Spectrum

R.Time:0.433(Scan#:)
MassPeaks:28
Segment 1 - Event 1

#	m/z	solute Inten	ative Intens
1	102.15	4385429	21.93
2	105.10	9714987	48.57
3	110.10	4366808	21.83
4	114.75	2231804	11.16
5	122.65	10073433	50.37
6	130.55	4621868	23.11
7	139.05	1369684	6.85
8	143.15	4442296	22.21
9	151.15	1695863	8.48
10	167.10	3695790	18.48
11	180.10	1467246	7.34
12	183.05	2992030	14.96
13	196.15	1832283	9.16
14	199.10	1058553	5.29
15	208.10	4964368	24.82
16	214.10	12945981	64.73
17	215.15	1660052	8.30
18	216.10	1066837	5.33
19	224.05	3194272	15.97

#	m/z	solute Inten	ative Intens
20	435.25	20000000	100.00
21	436.30	6408858	32.04
22	437.30	2080486	10.40
23	571.20	1200278	6.00
24	931.45	9702024	48.51
25	932.45	5358266	26.79
26	933.45	6513540	32.57
27	934.40	2830446	14.15
28	935.35	1057383	5.29

4B

SHIMADZU LabSolutions

Analysis Report

<Sample Information>

Sample Name	: 220516 NJ 1911		
Sample ID	: 220516 NJ 1911		
Data Filename	: 220516 NJ 1911.lcd		
Method Filename	: NNDKG.lcm		
Batch Filename	:		
Vial #	: 1-1	Sample Type	: Unknown
Injection Volume	: 1 uL		
Date Acquired	: 16/05/2022 12:13:52	Acquired by	: System Administrator
Date Processed	: 16/05/2022 12:14:48	Processed by	: System Administrator

MS Spectrum

R.Time:0.433(Scan#:)
MassPeaks:4
Segment 1 - Event 1

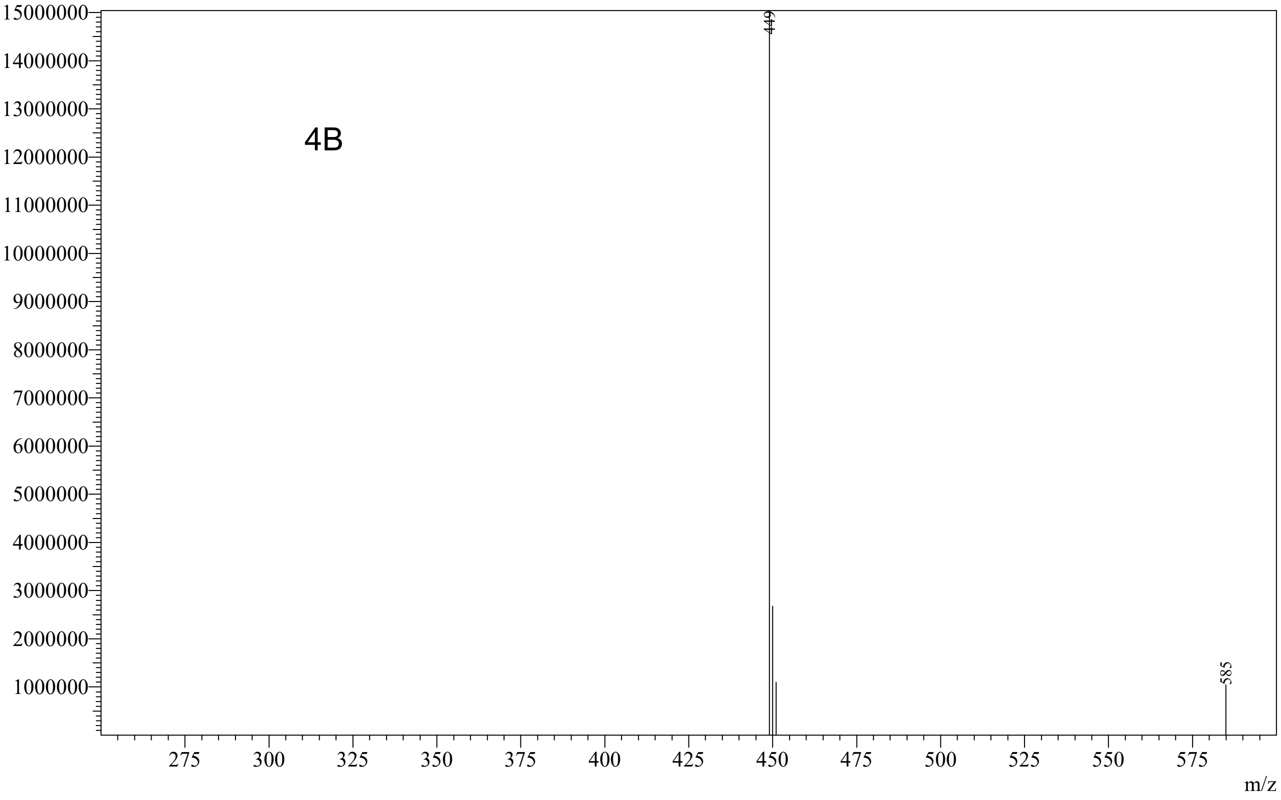

MS Spectrum

R.Time:0.433(Scan#:)
MassPeaks:28
Segment 1 - Event 1

#	m/z	solute Inten	ative Inten
1	102.15	4385429	21.93
2	105.10	9714987	48.57
3	110.10	4366808	21.83
4	114.75	2231804	11.16
5	122.65	10073433	50.37
6	130.55	4621868	23.11
7	139.05	1369684	6.85
8	143.15	4442296	22.21
9	151.15	1695863	8.48
10	167.10	3695790	18.48
11	180.10	1467246	7.34
12	183.05	2992030	14.96
13	196.15	1832283	9.16
14	199.10	1058553	5.29
15	208.10	4964368	24.82
16	214.10	12945981	64.73
17	215.15	1660052	8.30
18	216.10	1066837	5.33
19	224.05	3194272	15.97

#	m/z	solute Inten	ative Intens
20	435.25	20000000	100.00
21	436.30	6408858	32.04
22	437.30	2080486	10.40
23	571.20	1200278	6.00
24	931.45	9702024	48.51
25	932.45	5358266	26.79
26	933.45	6513540	32.57
27	934.40	2830446	14.15
28	935.35	1057383	5.29

4C

SHIMADZU LabSolutions

Analysis Report

<Sample Information>

Sample Name	: 220516 NJ 1692		
Sample ID	: 220516 NJ 1692		
Data Filename	: 220516 NJ 1692.lcd		
Method Filename	: NNDKG.lcm		
Batch Filename	:		
Vial #	: 1-1	Sample Type	: Unknown
Injection Volume	: 1 uL		
Date Acquired	: 16/05/2022 13:05:28	Acquired by	: System Administrator
Date Processed	: 16/05/2022 13:06:24	Processed by	: System Administrator

MS Spectrum

R.Time:0.433(Scan#:)
MassPeaks:5
Segment 1 - Event 1

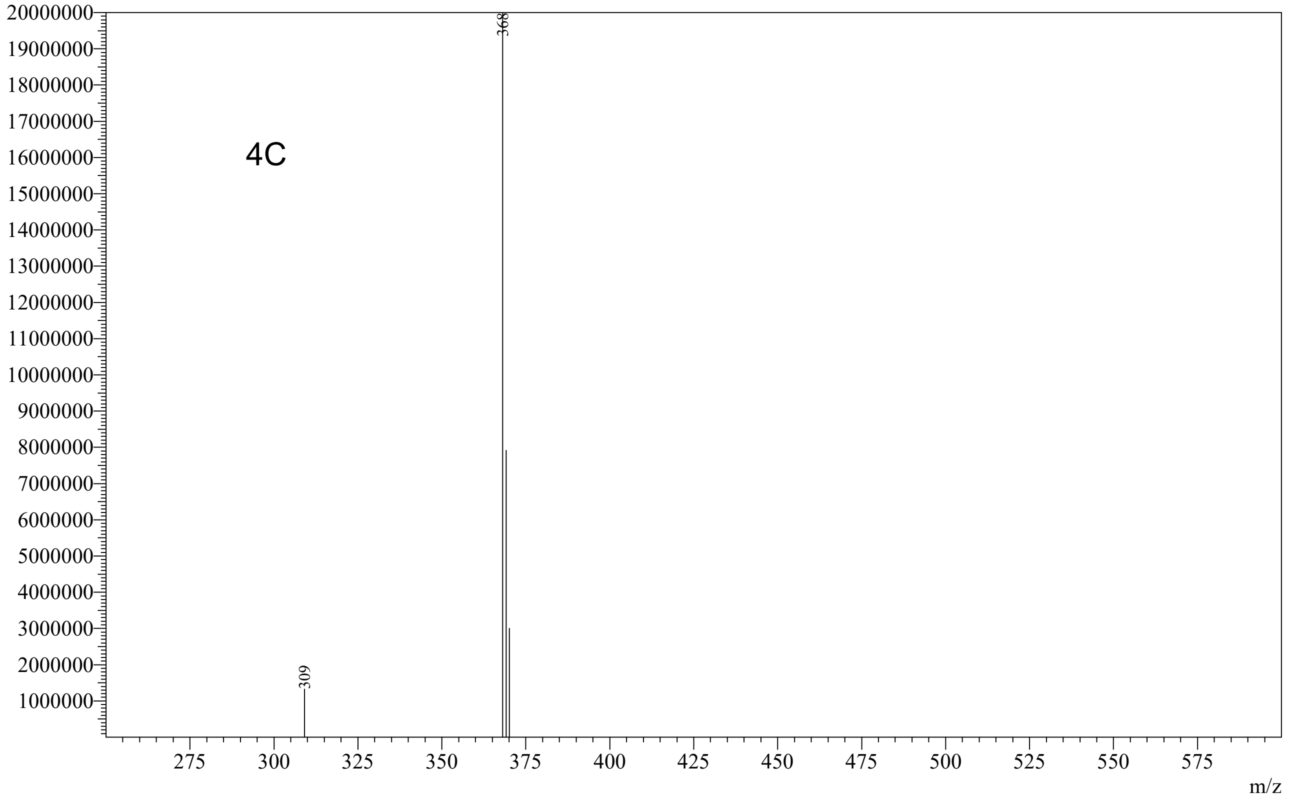

MS Spectrum

R.Time:0.433(Scan#:)
MassPeaks:28
Segment 1 - Event 1

#	m/z	solute Inten	ative Intens
1	102.15	4385429	21.93
2	105.10	9714987	48.57
3	110.10	4366808	21.83
4	114.75	2231804	11.16
5	122.65	10073433	50.37
6	130.55	4621868	23.11
7	139.05	1369684	6.85
8	143.15	4442296	22.21
9	151.15	1695863	8.48
10	167.10	3695790	18.48
11	180.10	1467246	7.34
12	183.05	2992030	14.96
13	196.15	1832283	9.16
14	199.10	1058553	5.29
15	208.10	4964368	24.82
16	214.10	12945981	64.73
17	215.15	1660052	8.30
18	216.10	1066837	5.33
19	224.05	3194272	15.97

#	m/z	solute Inten	ative Inten
20	435.25	20000000	100.00
21	436.30	6408858	32.04
22	437.30	2080486	10.40
23	571.20	1200278	6.00
24	931.45	9702024	48.51
25	932.45	5358266	26.79
26	933.45	6513540	32.57
27	934.40	2830446	14.15
28	935.35	1057383	5.29

4D

SHIMADZU LabSolutions

Analysis Report

<Sample Information>

Sample Name	: 220516 NJ 1913		
Sample ID	: 220516 NJ 1913		
Data Filename	: 220516 NJ 1913.lcd		
Method Filename	: NNDKG.lcm		
Batch Filename	:		
Vial #	: 1-1	Sample Type	: Unknown
Injection Volume	: 1 uL		
Date Acquired	: 16/05/2022 12:35:26	Acquired by	: System Administrator
Date Processed	: 16/05/2022 12:36:22	Processed by	: System Administrator

MS Spectrum

R.Time:0.433(Scan#:)
MassPeaks:7
Segment 1 - Event 1

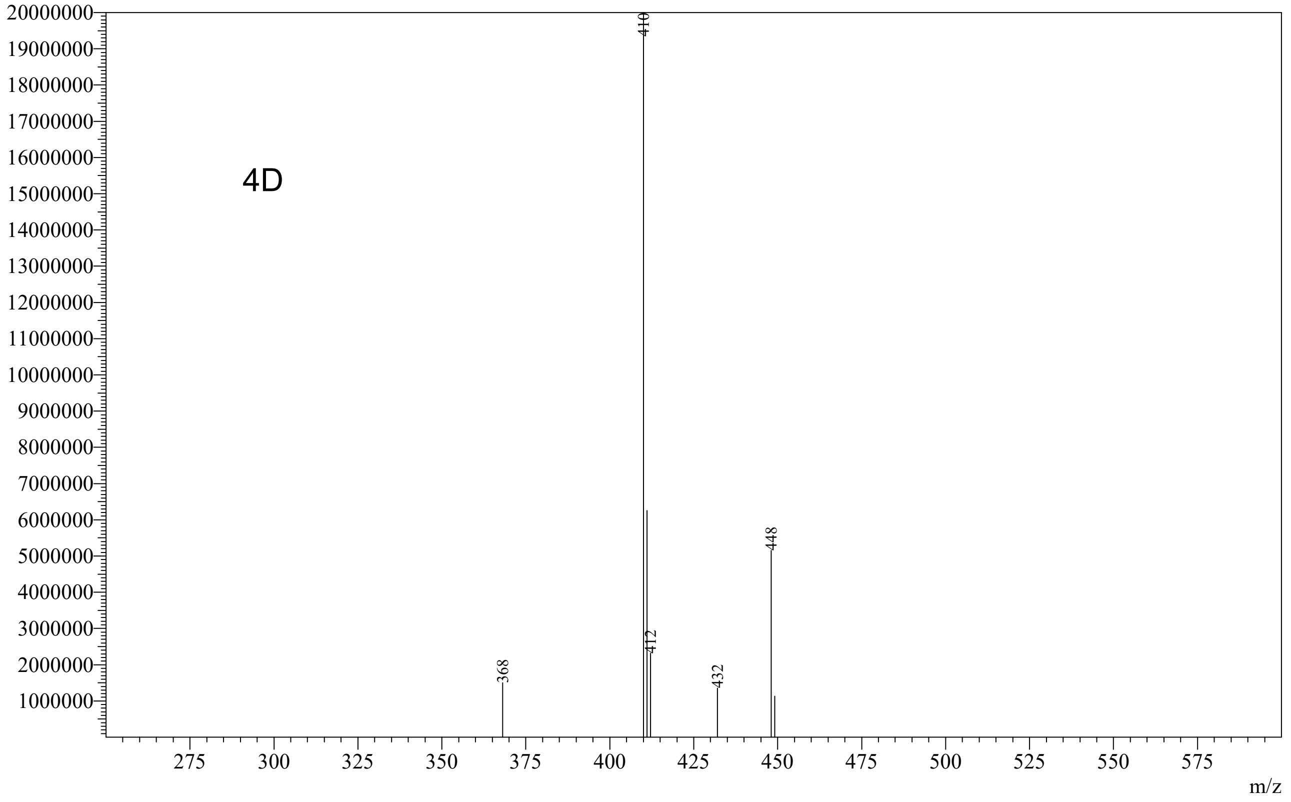

MS Spectrum

R.Time:0.433(Scan#:)
MassPeaks:28
Segment 1 - Event 1

#	m/z	solute Inten	ative Intens
1	102.15	4385429	21.93
2	105.10	9714987	48.57
3	110.10	4366808	21.83
4	114.75	2231804	11.16
5	122.65	10073433	50.37
6	130.55	4621868	23.11
7	139.05	1369684	6.85
8	143.15	4442296	22.21
9	151.15	1695863	8.48
10	167.10	3695790	18.48
11	180.10	1467246	7.34
12	183.05	2992030	14.96
13	196.15	1832283	9.16
14	199.10	1058553	5.29
15	208.10	4964368	24.82
16	214.10	12945981	64.73
17	215.15	1660052	8.30
18	216.10	1066837	5.33
19	224.05	3194272	15.97

#	m/z	solute Inten	ative Intens
20	435.25	20000000	100.00
21	436.30	6408858	32.04
22	437.30	2080486	10.40
23	571.20	1200278	6.00
24	931.45	9702024	48.51
25	932.45	5358266	26.79
26	933.45	6513540	32.57
27	934.40	2830446	14.15
28	935.35	1057383	5.29

4E

SHIMADZU LabSolutions

Analysis Report

<Sample Information>

Sample Name	: 220516 NJ 1933		
Sample ID	: 220516 NJ 1933		
Data Filename	: 220516 NJ 1933.lcd		
Method Filename	: NNDKG.lcm		
Batch Filename	:		
Vial #	: 1-1	Sample Type	: Unknown
Injection Volume	: 1 uL		
Date Acquired	: 16/05/2022 12:18:54	Acquired by	: System Administrator
Date Processed	: 16/05/2022 12:19:50	Processed by	: System Administrator

MS Spectrum

R.Time:0.433(Scan#:)
MassPeaks:10
Segment 1 - Event 1

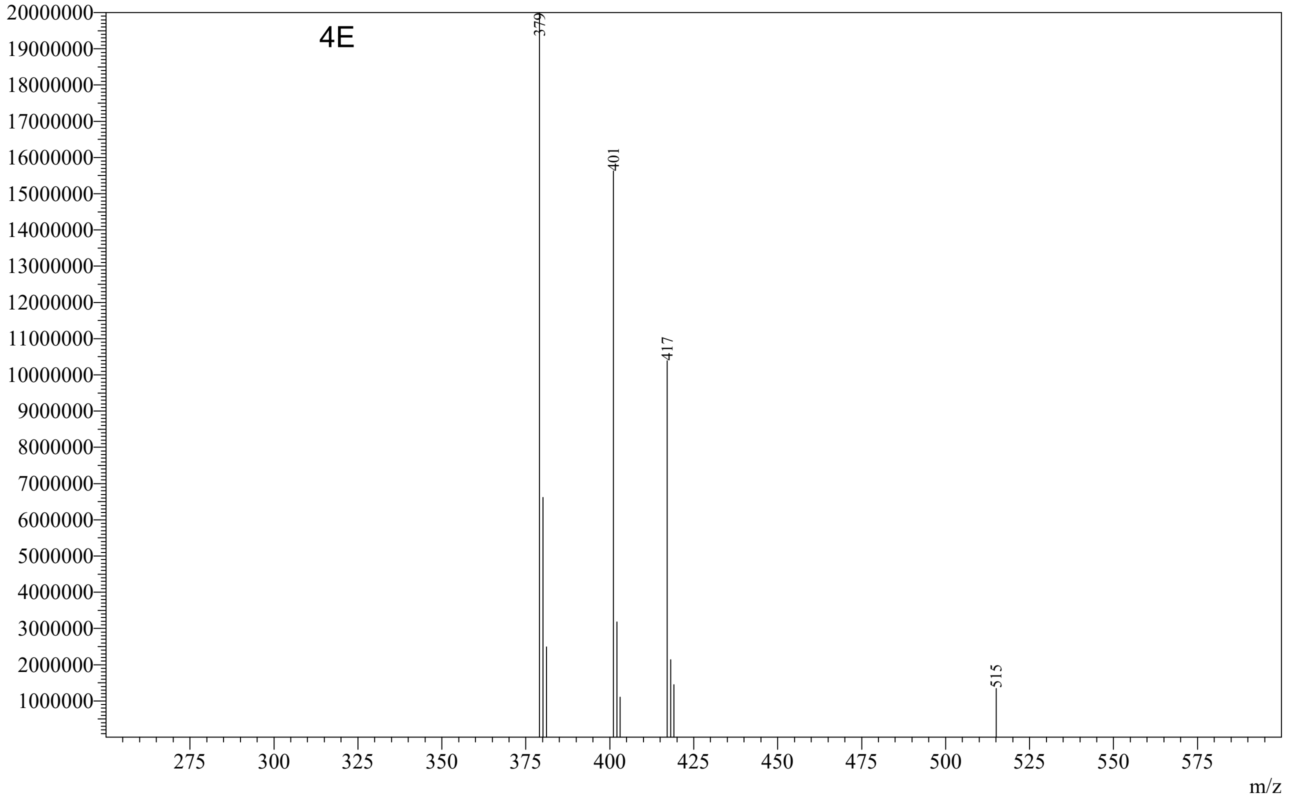

MS Spectrum

R.Time:0.433(Scan#:)
MassPeaks:28
Segment 1 - Event 1

#	m/z	solute Inten	ative Intens
1	102.15	4385429	21.93
2	105.10	9714987	48.57
3	110.10	4366808	21.83
4	114.75	2231804	11.16
5	122.65	10073433	50.37
6	130.55	4621868	23.11
7	139.05	1369684	6.85
8	143.15	4442296	22.21
9	151.15	1695863	8.48
10	167.10	3695790	18.48
11	180.10	1467246	7.34
12	183.05	2992030	14.96
13	196.15	1832283	9.16
14	199.10	1058553	5.29
15	208.10	4964368	24.82
16	214.10	12945981	64.73
17	215.15	1660052	8.30
18	216.10	1066837	5.33
19	224.05	3194272	15.97

#	m/z	solute Inten	ative Intens
20	435.25	20000000	100.00
21	436.30	6408858	32.04
22	437.30	2080486	10.40
23	571.20	1200278	6.00
24	931.45	9702024	48.51
25	932.45	5358266	26.79
26	933.45	6513540	32.57
27	934.40	2830446	14.15
28	935.35	1057383	5.29

4F

SHIMADZU LabSolutions

Analysis Report

<Sample Information>

Sample Name	: 220516 NJ 1897		
Sample ID	: 220516 NJ 1897		
Data Filename	: 220516 NJ 1897.lcd		
Method Filename	: NNDKG.lcm		
Batch Filename	:		
Vial #	: 1-1	Sample Type	: Unknown
Injection Volume	: 1 uL		
Date Acquired	: 16/05/2022 12:41:43	Acquired by	: System Administrator
Date Processed	: 16/05/2022 12:42:38	Processed by	: System Administrator

MS Spectrum

R.Time:0.433(Scan#:)
MassPeaks:13
Segment 1 - Event 1

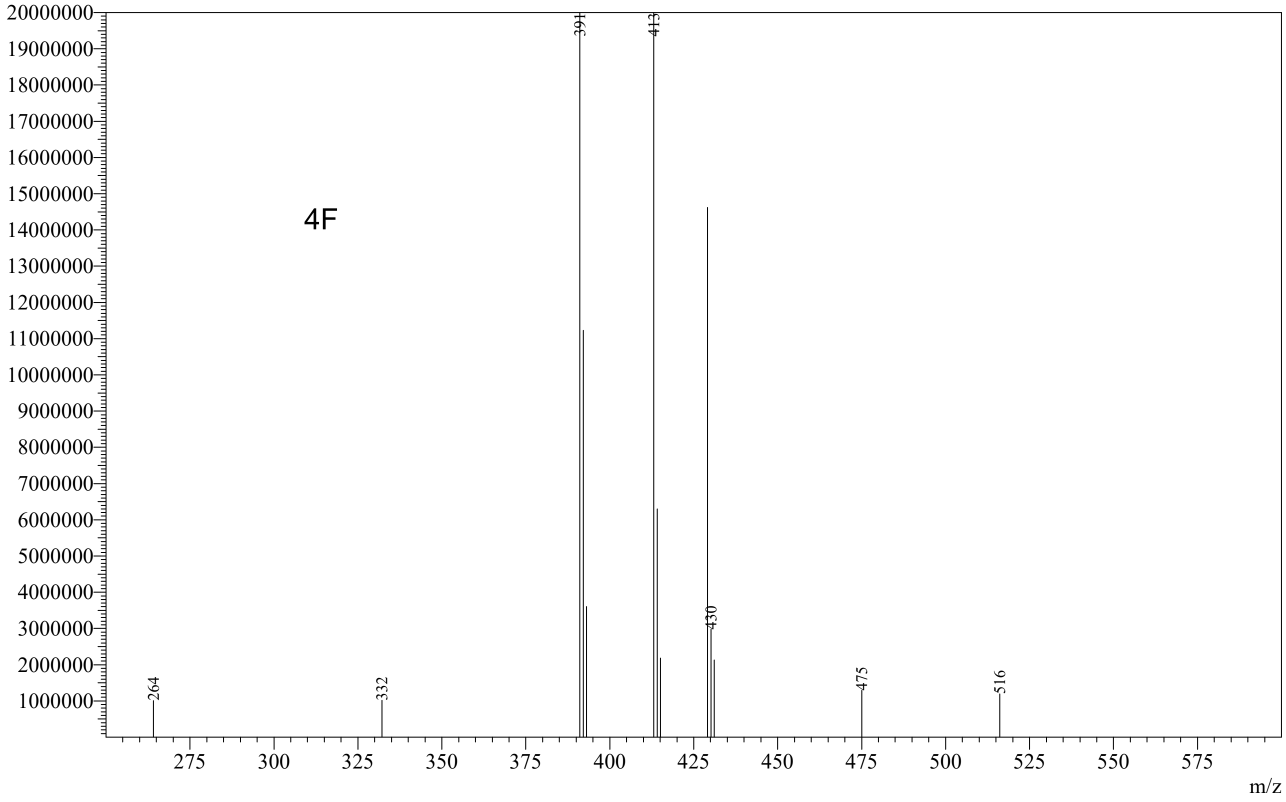

MS Spectrum

R.Time:0.433(Scan#:)
MassPeaks:28
Segment 1 - Event 1

#	m/z	solute Inten	ative Inten
1	102.15	4385429	21.93
2	105.10	9714987	48.57
3	110.10	4366808	21.83
4	114.75	2231804	11.16
5	122.65	10073433	50.37
6	130.55	4621868	23.11
7	139.05	1369684	6.85
8	143.15	4442296	22.21
9	151.15	1695863	8.48
10	167.10	3695790	18.48
11	180.10	1467246	7.34
12	183.05	2992030	14.96
13	196.15	1832283	9.16
14	199.10	1058553	5.29
15	208.10	4964368	24.82
16	214.10	12945981	64.73
17	215.15	1660052	8.30
18	216.10	1066837	5.33
19	224.05	3194272	15.97

#	m/z	solute Inten	ative Inten
20	435.25	20000000	100.00
21	436.30	6408858	32.04
22	437.30	2080486	10.40
23	571.20	1200278	6.00
24	931.45	9702024	48.51
25	932.45	5358266	26.79
26	933.45	6513540	32.57
27	934.40	2830446	14.15
28	935.35	1057383	5.29

4G

SHIMADZU LabSolutions

Analysis Report

<Sample Information>

Sample Name	: 220516 NJ 1898		
Sample ID	: 220516 NJ 1898		
Data Filename	: 220516 NJ 1898.lcd		
Method Filename	: NNDKG.lcm		
Batch Filename	:		
Vial #	: 1-1	Sample Type	: Unknown
Injection Volume	: 1 uL		
Date Acquired	: 16/05/2022 13:07:35	Acquired by	: System Administrator
Date Processed	: 16/05/2022 13:08:31	Processed by	: System Administrator

MS Spectrum

R.Time:0.433(Scan#:)
MassPeaks:21
Segment 1 - Event 1

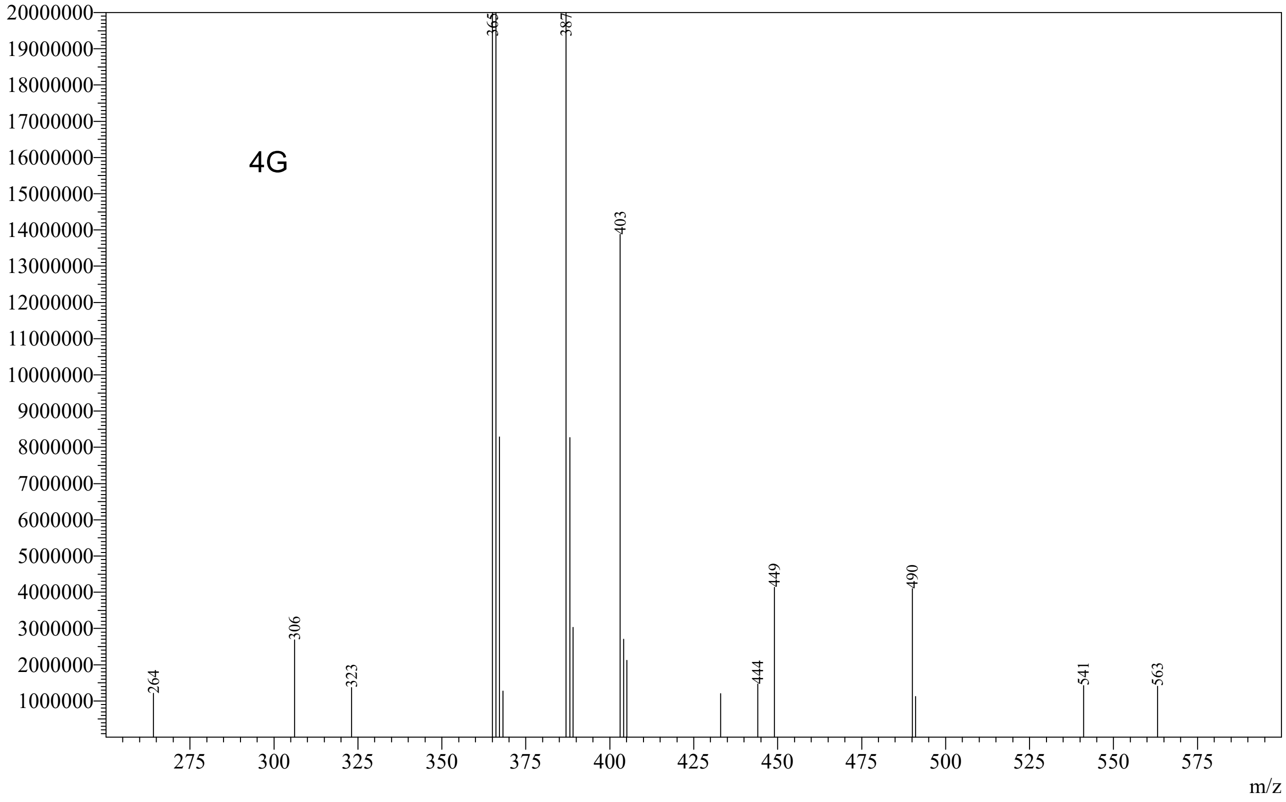

MS Spectrum

R.Time:0.433(Scan#:)
MassPeaks:28
Segment 1 - Event 1

#	m/z	solute Inten	ative Inten
1	102.15	4385429	21.93
2	105.10	9714987	48.57
3	110.10	4366808	21.83
4	114.75	2231804	11.16
5	122.65	10073433	50.37
6	130.55	4621868	23.11
7	139.05	1369684	6.85
8	143.15	4442296	22.21
9	151.15	1695863	8.48
10	167.10	3695790	18.48
11	180.10	1467246	7.34
12	183.05	2992030	14.96
13	196.15	1832283	9.16
14	199.10	1058553	5.29
15	208.10	4964368	24.82
16	214.10	12945981	64.73
17	215.15	1660052	8.30
18	216.10	1066837	5.33
19	224.05	3194272	15.97

#	m/z	solute Inten	ative Intens
20	435.25	20000000	100.00
21	436.30	6408858	32.04
22	437.30	2080486	10.40
23	571.20	1200278	6.00
24	931.45	9702024	48.51
25	932.45	5358266	26.79
26	933.45	6513540	32.57
27	934.40	2830446	14.15
28	935.35	1057383	5.29

4H

SHIMADZU LabSolutions

Analysis Report

<Sample Information>

Sample Name : 220516 NJ 1912
Sample ID : 220516 NJ 1912
Data Filename : 220516 NJ 1912.lcd
Method Filename : NNDKG.lcm
Batch Filename :
Vial # : 1-1 Sample Type : Unknown
Injection Volume : 1 uL
Date Acquired : 16/05/2022 12:31:19 Acquired by : System Administrator
Date Processed : 16/05/2022 12:32:15 Processed by : System Administrator

MS Spectrum

R.Time:0.433(Scan#:)
MassPeaks:12
Segment 1 - Event 1

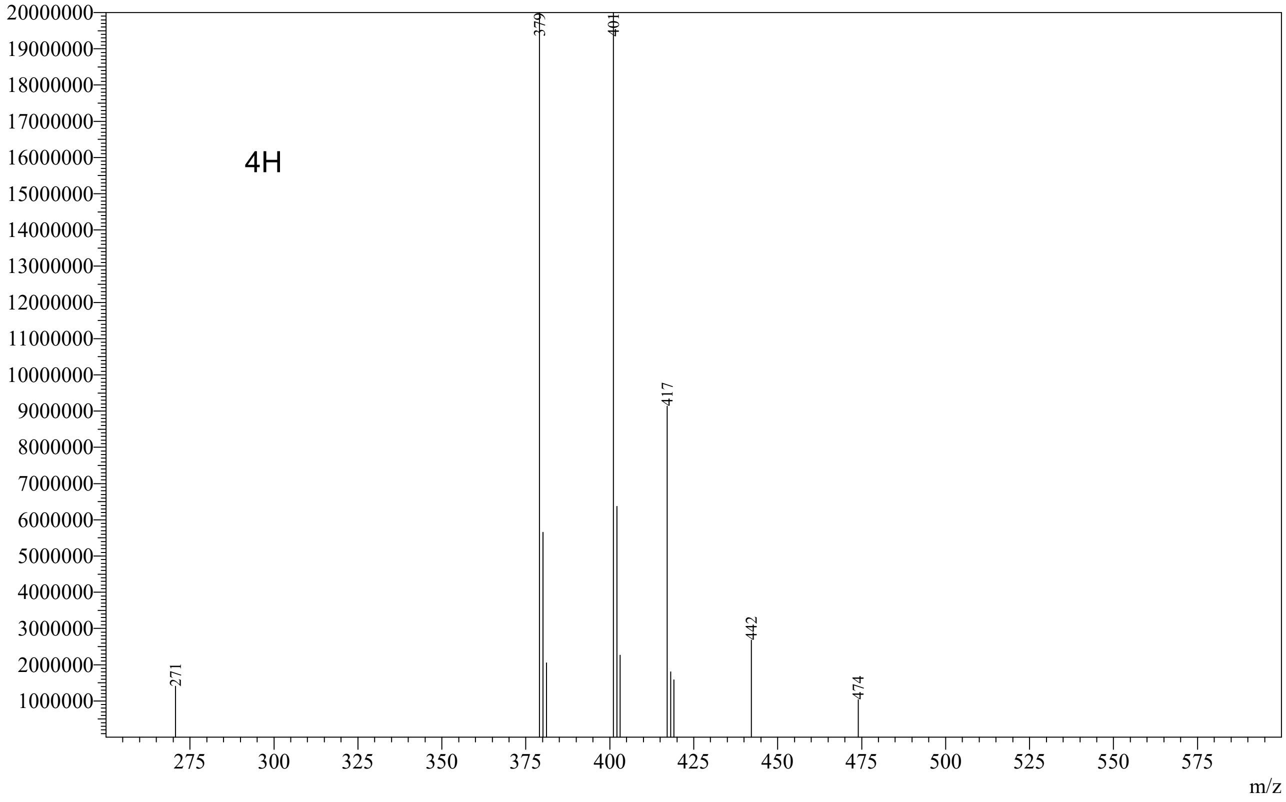

MS Spectrum

R.Time:0.433(Scan#:)
MassPeaks:28
Segment 1 - Event 1

#	m/z	solute Inten	ative Intens
1	102.15	4385429	21.93
2	105.10	9714987	48.57
3	110.10	4366808	21.83
4	114.75	2231804	11.16
5	122.65	10073433	50.37
6	130.55	4621868	23.11
7	139.05	1369684	6.85
8	143.15	4442296	22.21
9	151.15	1695863	8.48
10	167.10	3695790	18.48
11	180.10	1467246	7.34
12	183.05	2992030	14.96
13	196.15	1832283	9.16
14	199.10	1058553	5.29
15	208.10	4964368	24.82
16	214.10	12945981	64.73
17	215.15	1660052	8.30
18	216.10	1066837	5.33
19	224.05	3194272	15.97

#	m/z	solute Inten	ative Intens
20	435.25	20000000	100.00
21	436.30	6408858	32.04
22	437.30	2080486	10.40
23	571.20	1200278	6.00
24	931.45	9702024	48.51
25	932.45	5358266	26.79
26	933.45	6513540	32.57
27	934.40	2830446	14.15
28	935.35	1057383	5.29

4l

SHIMADZU LabSolutions

Analysis Report

<Sample Information>

Sample Name	: 220516 NJ 1916		
Sample ID	: 220516 NJ 1916		
Data Filename	: 220516 NJ 1916.lcd		
Method Filename	: NNDKG.lcm		
Batch Filename	:		
Vial #	: 1-1	Sample Type	: Unknown
Injection Volume	: 1 uL		
Date Acquired	: 16/05/2022 12:49:48	Acquired by	: System Administrator
Date Processed	: 16/05/2022 12:50:43	Processed by	: System Administrator

MS Spectrum

R.Time:0.433(Scan#:)
MassPeaks:22
Segment 1 - Event 1

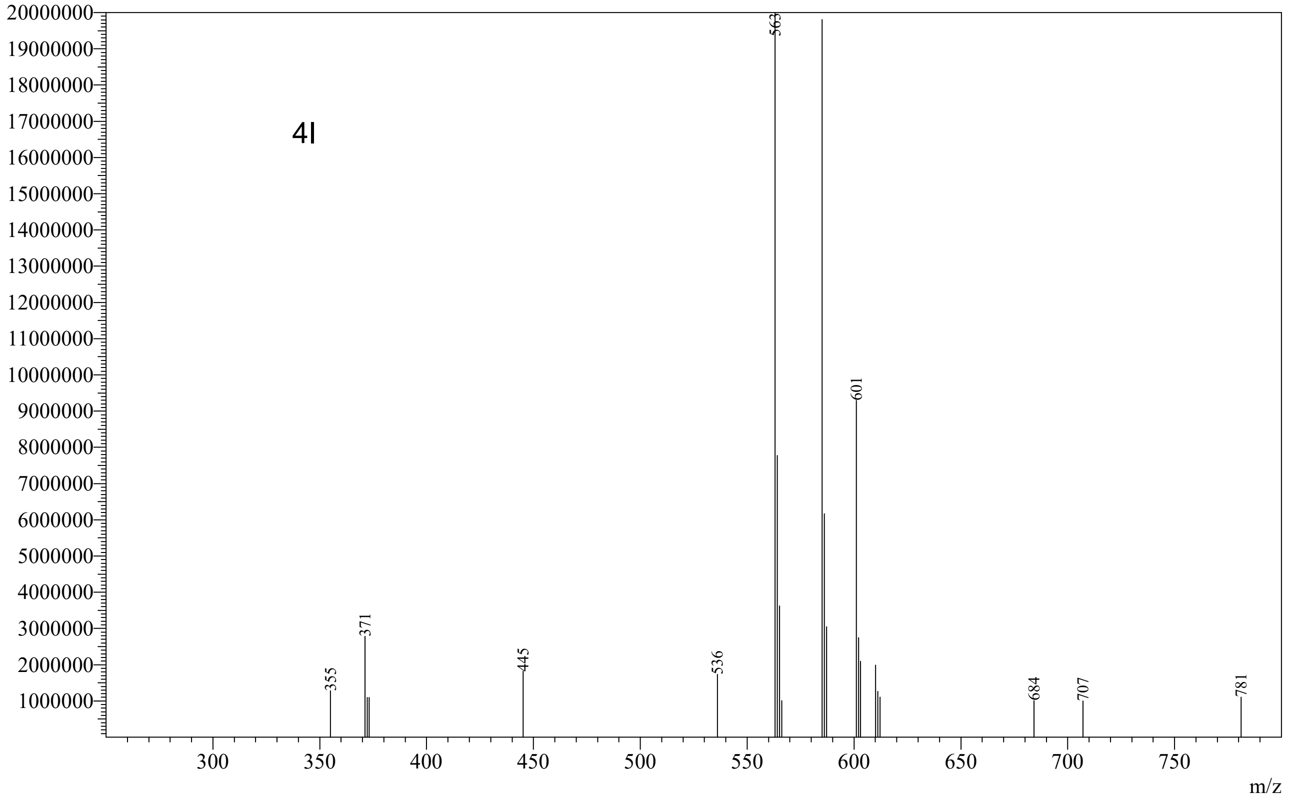

MS Spectrum

R.Time:0.433(Scan#:)
MassPeaks:28
Segment 1 - Event 1

#	m/z	solute Inten	ative Intens
1	102.15	4385429	21.93
2	105.10	9714987	48.57
3	110.10	4366808	21.83
4	114.75	2231804	11.16
5	122.65	10073433	50.37
6	130.55	4621868	23.11
7	139.05	1369684	6.85
8	143.15	4442296	22.21
9	151.15	1695863	8.48
10	167.10	3695790	18.48
11	180.10	1467246	7.34
12	183.05	2992030	14.96
13	196.15	1832283	9.16
14	199.10	1058553	5.29
15	208.10	4964368	24.82
16	214.10	12945981	64.73
17	215.15	1660052	8.30
18	216.10	1066837	5.33
19	224.05	3194272	15.97

#	m/z	solute Inten	ative Intens
20	435.25	20000000	100.00
21	436.30	6408858	32.04
22	437.30	2080486	10.40
23	571.20	1200278	6.00
24	931.45	9702024	48.51
25	932.45	5358266	26.79
26	933.45	6513540	32.57
27	934.40	2830446	14.15
28	935.35	1057383	5.29

4J

SHIMADZU LabSolutions

Analysis Report

<Sample Information>

Sample Name	: 220516 NJ 1922		
Sample ID	: 220516 NJ 1922		
Data Filename	: 220516 NJ 1924.lcd		
Method Filename	: NNDKG.lcm		
Batch Filename	:		
Vial #	: 1-1	Sample Type	: Unknown
Injection Volume	: 1 uL		
Date Acquired	: 16/05/2022 12:24:19	Acquired by	: System Administrator
Date Processed	: 16/05/2022 12:25:15	Processed by	: System Administrator

MS Spectrum

R.Time:0.433(Scan#:)
MassPeaks:9
Segment 1 - Event 1

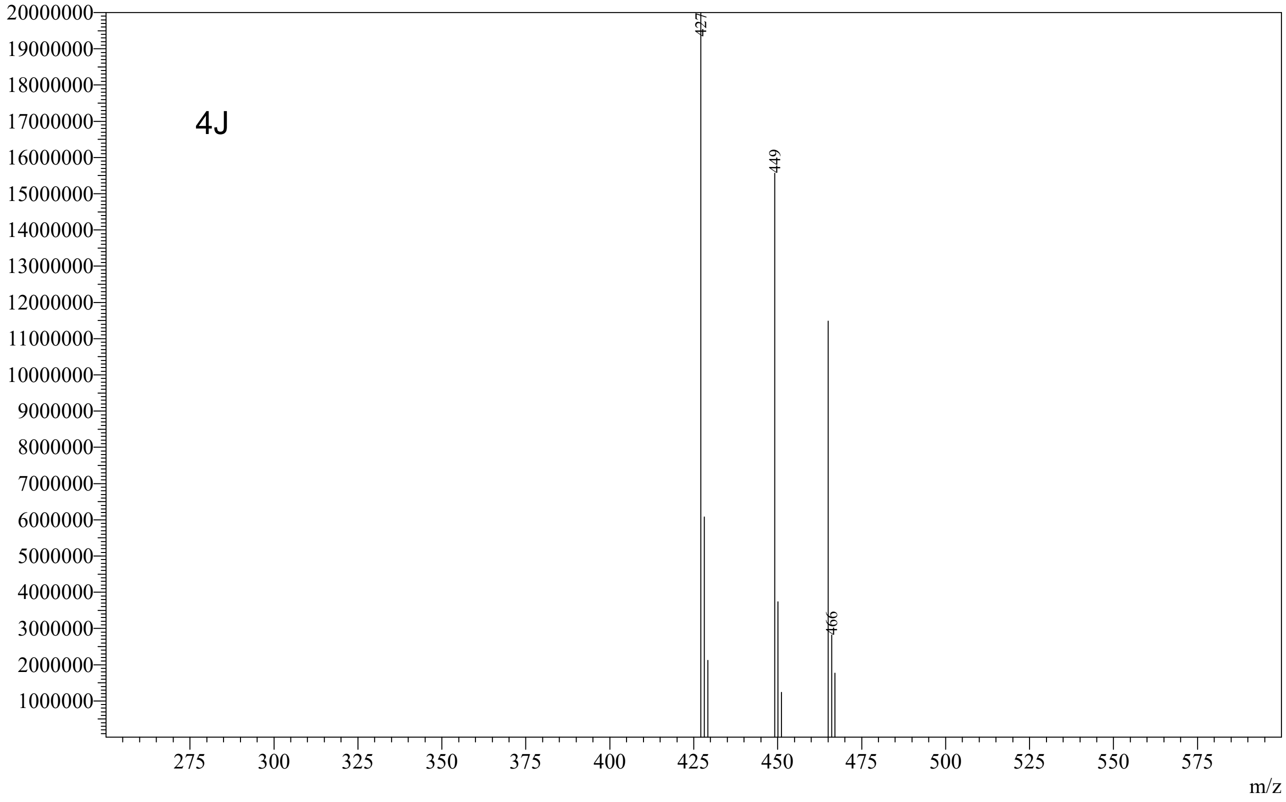

MS Spectrum

R.Time:0.433(Scan#:)
MassPeaks:28
Segment 1 - Event 1

#	m/z	solute Inten	ative Intens
1	102.15	4385429	21.93
2	105.10	9714987	48.57
3	110.10	4366808	21.83
4	114.75	2231804	11.16
5	122.65	10073433	50.37
6	130.55	4621868	23.11
7	139.05	1369684	6.85
8	143.15	4442296	22.21
9	151.15	1695863	8.48
10	167.10	3695790	18.48
11	180.10	1467246	7.34
12	183.05	2992030	14.96
13	196.15	1832283	9.16
14	199.10	1058553	5.29
15	208.10	4964368	24.82
16	214.10	12945981	64.73
17	215.15	1660052	8.30
18	216.10	1066837	5.33
19	224.05	3194272	15.97

#	m/z	solute Inten	ative Inten
20	435.25	20000000	100.00
21	436.30	6408858	32.04
22	437.30	2080486	10.40
23	571.20	1200278	6.00
24	931.45	9702024	48.51
25	932.45	5358266	26.79
26	933.45	6513540	32.57
27	934.40	2830446	14.15
28	935.35	1057383	5.29

4K

SHIMADZU LabSolutions

Analysis Report

<Sample Information>

Sample Name	: 220516 NJ 1941		
Sample ID	: 220516 NJ 1941		
Data Filename	: 220516 NJ 1941.lcd		
Method Filename	: NNDKG.lcm		
Batch Filename	:		
Vial #	: 1-1	Sample Type	: Unknown
Injection Volume	: 1 uL		
Date Acquired	: 16/05/2022 12:39:37	Acquired by	: System Administrator
Date Processed	: 16/05/2022 12:40:32	Processed by	: System Administrator

MS Spectrum

R.Time:0.433(Scan#:)
MassPeaks:12
Segment 1 - Event 1

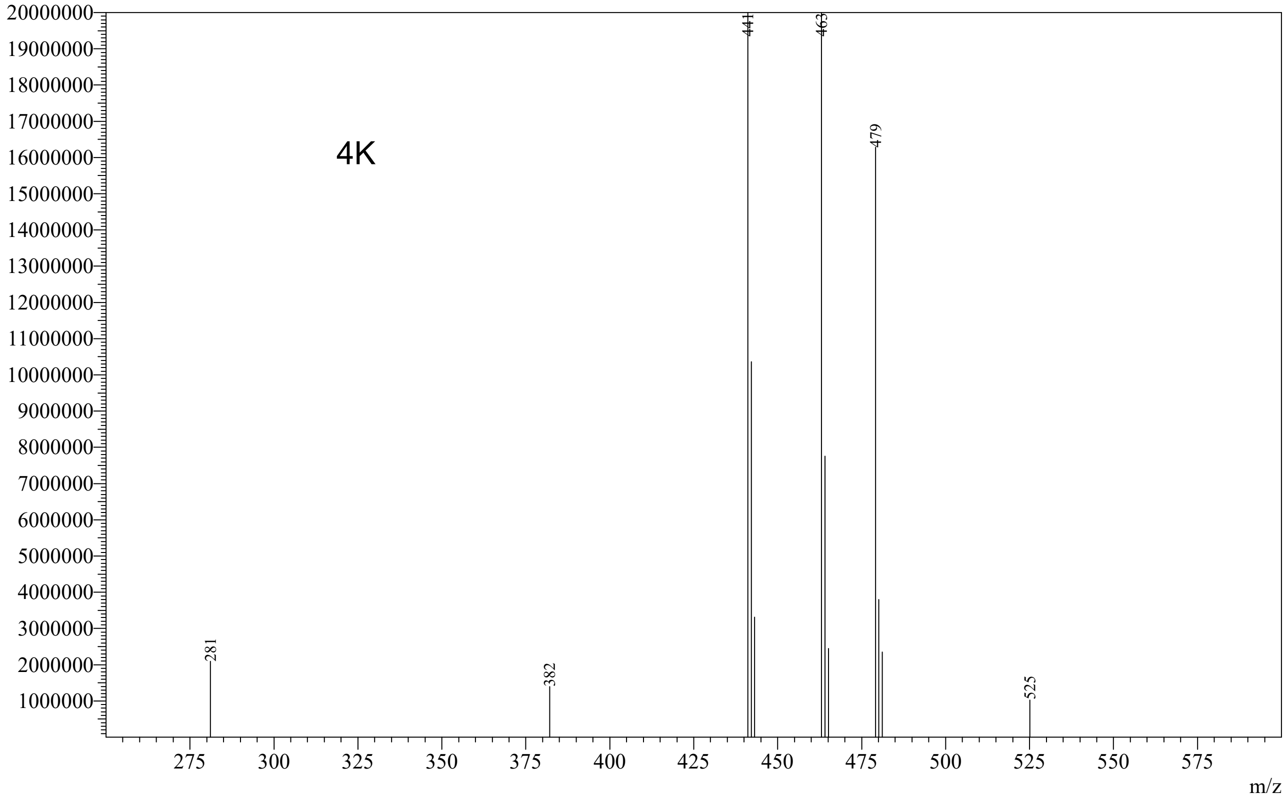

MS Spectrum

R.Time:0.433(Scan#:)
MassPeaks:28
Segment 1 - Event 1

#	m/z	solute Inten	ative Intens
1	102.15	4385429	21.93
2	105.10	9714987	48.57
3	110.10	4366808	21.83
4	114.75	2231804	11.16
5	122.65	10073433	50.37
6	130.55	4621868	23.11
7	139.05	1369684	6.85
8	143.15	4442296	22.21
9	151.15	1695863	8.48
10	167.10	3695790	18.48
11	180.10	1467246	7.34
12	183.05	2992030	14.96
13	196.15	1832283	9.16
14	199.10	1058553	5.29
15	208.10	4964368	24.82
16	214.10	12945981	64.73
17	215.15	1660052	8.30
18	216.10	1066837	5.33
19	224.05	3194272	15.97

#	m/z	solute Inten	ative Inten
20	435.25	20000000	100.00
21	436.30	6408858	32.04
22	437.30	2080486	10.40
23	571.20	1200278	6.00
24	931.45	9702024	48.51
25	932.45	5358266	26.79
26	933.45	6513540	32.57
27	934.40	2830446	14.15
28	935.35	1057383	5.29
